# Prioritising attributes for tuberculosis preventive treatment regimens: a modelling analysis

**DOI:** 10.1186/s12916-022-02378-1

**Published:** 2022-05-18

**Authors:** Juan F. Vesga, Christian Lienhardt, Placide Nsengiyumva, Jonathon R. Campbell, Olivia Oxlade, Saskia den Boon, Dennis Falzon, Kevin Schwartzman, Gavin Churchyard, Nimalan Arinaminpathy

**Affiliations:** 1 Department of Infectious Disease Epidemiology, Faculty of Medicine, Imperial College London, London, UK; 2 Institut de recherche pour le développement, Montpellier, France; 3 McGill International Tuberculosis Centre, McGill University, Montreal, Canada; 4 Global TB Programme, World Health Organization, Geneva, Switzerland; 5 The Aurum Institute, Parktown, South Africa; 6 School of Public Health, University of Witwatersrand, Johannesburg, South Africa

**Keywords:** Tuberculosis, Preventive therapy, Mathematical modelling

## Abstract

**Background:**

Recent years have seen important improvements in available preventive treatment regimens for tuberculosis (TB), and research is ongoing to develop these further. To assist with the formulation of target product profiles for future regimens, we examined which regimen properties would be most influential in the epidemiological impact of preventive treatment.

**Methods:**

Following expert consultation, we identified 5 regimen properties relevant to the incidence-reducing impact of a future preventive treatment regimen: regimen duration, efficacy, ease-of-adherence (treatment completion rates in programmatic conditions), forgiveness to non-completion and the barrier to developing rifampicin resistance during treatment. For each regimen property, we elicited expert input for minimally acceptable and optimal (ideal-but-feasible) performance scenarios for future regimens. Using mathematical modelling, we then examined how each regimen property would influence the TB incidence reduction arising from full uptake of future regimens according to current WHO guidelines, in four countries: South Africa, Kenya, India and Brazil.

**Results:**

Of all regimen properties, efficacy is the single most important predictor of epidemiological impact, while ease-of-adherence plays an important secondary role. These results are qualitatively consistent across country settings; sensitivity analyses show that these results are also qualitatively robust to a range of model assumptions, including the mechanism of action of future preventive regimens.

**Conclusions:**

As preventive treatment regimens against TB continue to improve, understanding the key drivers of epidemiological impact can assist in guiding further development. By meeting these key targets, future preventive treatment regimens could play a critical role in global efforts to end TB.

**Supplementary Information:**

The online version contains supplementary material available at 10.1186/s12916-022-02378-1.

## Background

Tuberculosis (TB) is a preventable disease [1–3]. WHO guidelines for preventive treatment have undergone several updates, most recently with the recommendation to include all household contacts of diagnosed TB cases, in addition to persons living with HIV [4]. However, there remain important challenges in reaching the expected population targets, particularly in expanding coverage amongst household contacts [5, 6]. In response to these challenges, recent years have seen important developments in the emergence of shorter, simpler regimens, with, for example, 6 months of daily isoniazid (6H) gradually being replaced by 3 months of once-weekly rifapentine and isoniazid (3HP) [7]. More recently, 1 month of isoniazid and rifapentine has been shown to be non-inferior to 9H [8], in adolescents and adults living with HIV (PLHIV).

In anticipation of continued research and development in this area, WHO recently published target product profiles (TPPs) for future preventive treatment regimens [9]. This document identified a series of essential and desirable attributes for future preventive treatment regimens, in order to optimize TB prevention worldwide. The need to prioritise systematically amongst these attributes raises some important questions: which would play the largest role in the epidemiological impact of a future preventive treatment regimen? How would these priorities vary in different countries and epidemiological contexts? In support of the WHO TPPs, we developed mathematical models of TB transmission dynamics to address these questions from a multi-country perspective.

## Methods

**Fig. 1:**
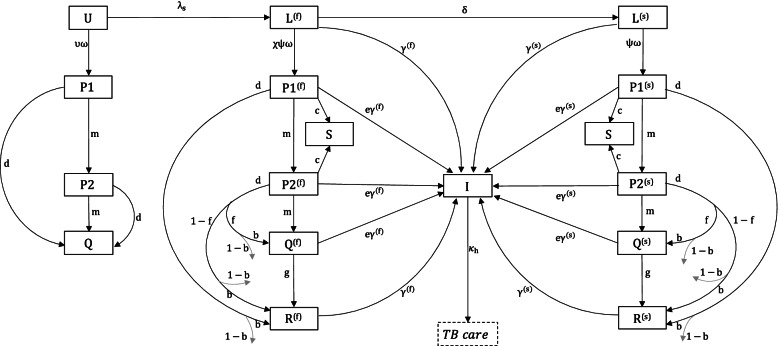
Schematic illustration of the model structure. For clarity, the figure concentrates on model compartments relevant to latent TB infection, its progression to active disease, and the effect of preventive treatment on these dynamics; further information on the care cascade for active TB disease (shown in the dotted rectangle) is provided in the supporting information. Each compartment shown here is further stratified by the HIV status. Amongst compartments relating to the natural history of TB, *U* denotes ‘uninfected’; *L*^(*f*)^denotes latent infection with ‘fast’ progression (in the first 2 years following infection); *L*^(*s*)^ denotes latent infection with ‘slow’ progression; and *I* denotes active, infectious TB. The modelled action of preventive treatment is as follows: *S* denotes individuals who are bacteriologically cured of infection as a result of the regimen; the parameter *c* governs the proportion cured in this way. *Q* denotes individuals with non-curative, post-regimen protection; the parameter *e* denotes the strength of non-curative protection, while *g* denotes its post-regimen durability. P1 and P2 represent individuals who are, respectively, undergoing the first and second halves of the regimen. Reinfection is possible for stages P1, P2, S, Q, at a reduced rate compared to R, given an assumed level of protection from preventive therapy. Reinfection is not shown for clarity, but is modelled by transitions from *P*1, *P*2, *Q* and *R* to their respective *f* states (e.g., *P*1^(*f*)^), and from *S* to *L*_*f*_. We assume that ‘forgiveness to non-completion’ (one of the regimen properties listed in Table 2) applies only to the latter, with the parameter *f* being the proportion interrupting treatment who nonetheless have the same outcomes as those completing treatment. *R* denotes individuals who have reverted to their pre-regimen state of TB infection, following decay of any post-regimen protection. *d* denotes the per-capita hazard of regimen interruption and thus governs ease-of-adherence, *b* directly models the drug-resistance barrier and *m* governs the regimen duration. These and remaining model parameters are as listed in Additional file 2: Table S2

We modelled the impact of future preventive treatment regimens in four countries: South Africa, Kenya, India, and Brazil. As shown in Table 1, South Africa and Kenya are high TB burden countries where HIV is a major driver of the TB epidemic; India is a high TB burden country where HIV does not play such a driving role; and Brazil is the country with the lowest incidence and mortality rates of the four. These countries also represent a range of socioeconomic settings, ranging from low-income to upper middle-income. For each country, we developed a deterministic, compartmental model of TB transmission dynamics, capturing HIV coinfection and the provision of antiretroviral therapy, as well as the implementation of preventive treatment amongst all-age household contacts of notified TB cases (see Fig. 1 for model schematic and  Additional file 1: Fig. S1 and Table S1 for further technical specifications, including model equations) The model incorporates the acquisition and transmission of rifampicin-resistant (RR) TB including multi-drug-resistant TB. It also incorporates the accelerating effect of HIV coinfection on TB progression. For simplicity, the model does not incorporate age structure, nor does it distinguish different forms of TB (e.g., smear status or extrapulmonary vs pulmonary TB), instead assuming an average infectiousness over these forms. For natural history parameters relating to TB infection, we drew from recent systematic reviews and modelling studies that identified models consistent with available data for TB progression (see Additional file 2: Table S2) [4, 6, 10–21]. As described below, we accommodated a range of possible mechanisms by which a future preventive treatment regimen would alter these dynamics.

**Table 1:** Comparison of countries in analysis. Shown are estimates for total annual TB incidence rates, and TB incidence among HIV+ only in 2019, drawn from the WHO Global TB Report 2019 [5]. While these estimates are shown for comparison, we also calibrated each model to country-specific data for TB mortality, notifications, the burden of rifampicin-resistant TB, and the coverage of antiretroviral therapy (ART) amongst those with PLHIV. Full country data used for calibration are listed in Additional file 4: Table S4

Country	TB Incidence per 100K (uncertainty range)	TB incidence per 100K (HIV+ only) (uncertainty range)
South Africa	615 (427–835)	357 (248–486)
Kenya	267 (163–396)	70 (43–104)
India	193 (121–384)	5.2 (3.6–7.2)
Brazil	46 (39–53)	5.1 (4.3–6)

Despite their simplicity and transparency, compartmental models do not lend themselves readily to modelling contacts of patients with TB disease. To address this challenge, our approach relies on recent data that suggests that the incidence rate in TB-infected household contacts of TB cases is 7–8 times greater than in the general population [22, 23]; this is because infections amongst contacts are more likely to have arisen from recent exposure than infections in the community. As described in Additional file 3 (Fig. S2, Table S3) [22, 24–26], our strategy is to model explicitly this disproportionate amount of recent infection in household contacts. An advantage of this approach, compared to previous modelling analyses of preventive treatment [27, 28], is that it allows us to incorporate the mechanisms by which future preventive regimens may act (mechanisms discussed further below).

### Calibration

For each country, we calibrated the model to country-specific estimates for TB incidence and mortality, incidence of RR-TB; the proportion of incident TB associated with concomitant HIV infection and the coverage of antiretroviral therapy (ART) amongst those living with HIV (PLHIV), as well as the coverage of current preventive treatment amongst those initiated on ART (Table 1). All of these estimates include uncertainty intervals: we used Bayesian melding [29, 30] to systematically propagate this uncertainty to model projections, assuming uniform priors on model parameters. Drawing 1000 posterior samples, we estimated Bayesian credible intervals (CrI) in model projections using 2.5th and 97.5th percentiles, and central estimates using the 50th percentile. Further details of model calibration can be found in Additional file 4 [31, 32].

### Modelling effects of preventive treatment

In each country, we modelled the potential impact of future preventive treatment regimens on TB incidence, assuming that the regimen is introduced in 2021 and scaled up in a linear way over the subsequent 3 years to cover all PLHIVs on ART (hereafter referred to simply as “PLHIV”) and all household contacts of notified TB cases (including both PLHIV and non-HIV population), with this coverage being maintained until 2035. To account for the overlap between household contacts and PLHIV, we assumed for simplicity that the proportion with HIV is the same in households as at the population level; thus, in the model, it is possible for PLHIV to be initiated on preventive therapy either through their HIV status, or through being identified as household contacts of diagnosed TB patients. Simulating epidemic trajectories to 2035, we assessed the reduction in cumulative incidence as a result of preventive treatment in both risk groups.

To specify regimen properties, we drew from ongoing expert and community consultations that were being conducted to inform the WHO TPPs [33]. In brief, these consultations aimed to identify key attributes for future regimens [9]. We summarised these attributes as 5 regimen properties that would be most relevant for epidemiological impact: efficacy, ease-of-adherence, forgiveness to regimen non-completion, regimen duration and barrier to developing rifampicin resistance (described in further detail below). It is helpful here to make a distinction between regimen ‘properties’ (as employed in the present analysis) and regimen ‘attributes’ (as identified in the TPPs). Although the former is based on the latter, they do not necessarily have a one-to-one correspondence: for instance, ease-of-adherence represents an amalgam of different attributes in the TPPs, e.g., pill burden and tolerability. Forgiveness for non-completion, while not mentioned explicitly in the TPPs, is included in the current analysis because of its potential impact on TB incidence. Hereafter, we focus on regimen ‘properties’ as the basis for analysis. For simplicity, we assumed that regimen properties are unaffected by HIV status, motivated by current preventive therapy regimens that show 60% efficacy in both HIV-positive and HIV-negative individuals [7, 34].

Table 2 lists these five regimen properties, together with scenarios—drawn from expert opinion—for ‘minimal’ and ‘optimal’ values. Here, the ‘minimal’ scenario describes the lowest performance threshold that future regimens would need to fulfil in order to be licensed and recommended, while the ‘optimal’ scenario represents performance parameters deemed ideal but achievable. Values for these parameters were initially provided by a core group of technical and scientific experts amongst those who contributed to the TPPs and subsequently validated when presenting modelling assumptions and results to the full group of experts (see [33] for a description of the group of experts).

**Table 2:** Modelled regimen properties and their minimal and optimal scenarios. While the full-target product profile published by WHO [9] lists an extended series of regimen attributes, for the purpose of the current modelling analysis, we distilled these to the limited set of properties shown here, that would be most relevant to the transmission impact of a future regimen. ‘Minimal’ and ‘Optimal’ values for regimen properties were identified through expert opinion from the experts involved in the WHO target product profiles: they represent the range between minimally acceptable performance parameters for a future preventive treatment regimen to be licensed and recommended, and on the other hand, ideal but achievable regimen properties. Footnotes: (i) In the main text, we assumed that regimens would have half the efficacy against rifampicin-resistant TB infection, compared to their efficacy against drug-sensitive infection: in sensitivity analysis, we modified this assumption to 25% and 75%. (ii) 100% efficacy is likely to be infeasible to be achieved in practice, but is stated here as an aspirational target. (iii) Ease-of-adherence incorporates a range of regimen properties listed in the WHO target product profiles, including pill burden, dosing frequency, and safety and tolerability. As discussed in the main text, further evidence is needed to understand quantitatively how each of these regimen properties drives ease-of-adherence. (iv) To avoid an overly optimistic role of forgiveness, we assumed in the main analysis that forgiveness only applies to those completing at least 50% of the regimen (and that any less is associated with essentially zero forgiveness). In sensitivity analysis, we adjusted this threshold to 25% and 75% (see Additional file 7: Table S9, and Fig. S14)

Regimen property	How it is quantified	Future PT regimen	
		Minimal	Optimal
Regimen duration	Duration of administration of the regimen (months)	3	1
Efficacy against drug-sensitive TB (modelled as emergent property of ‘hidden’ mechanistic properties including proportion bacteriologically cured, and strength and durability of non-curative protection)	Efficacy measured as reduction in incidence among those with TB infection that would be observed under trial conditions at two-year post-regimen follow-up, in a cohort receiving the regimen vs a hypothetical cohort receiving placebo (see Methods for further technical details of cohort modelling) (i)	70%	100% (ii)
Ease-of-adherence	Proportion successfully completing the regimen under programmatic conditions (iii)	80%	90%
Forgiveness	Amongst those completing at least half of the regimen before interrupting, the proportion that nonetheless have the same outcomes as those completing the full regimen (iv)	50%	80%
Barrier to resistance	Amongst those having rifampicin-sensitive infection, the proportion that do not develop resistant infection as a result of preventive treatment	95%	100%

Some of these properties bear mention. First, efficacy is typically measured in trial conditions as the reduction in TB incidence over 2 or more years of longitudinal follow-up in a cohort receiving the regimen under study, compared to placebo in early trials, or more recently to standard of care using regimens of known efficacy [7, 8]. Due to its pharmacokinetic/pharmacodynamic properties, the effect of preventive treatment could range from partially clearing infection, allowing future reactivation (although at a lower risk than in the absence of treatment) to fully sterilising infection (i.e., bacteriological cure) [27, 35, 36]. Accordingly, we distinguished two types of protection: ‘non-curative’ protection as causing only partial reduction in incidence, and having finite duration; and ‘cure’ as a permanent, 100% reduction in incidence in the absence of reinfection. In the case of non-curative protection, the duration is expected to be longer than the duration of the regimen itself, owing to the bactericidal effect of the regimen, and its synergistic interactions with host immunity-mediated control of infection [37]. Taken together, we thus characterised the pharmacokinetic/pharmacodynamic effect of any future regimen in terms of three mechanistic parameters: (i) amongst those completing the regimen, the proportion bacteriologically cured; (ii) amongst those not cured, the reduction in the risk of reactivation arising from non-curative protection; (iii) the average duration, following completion of the regimen, during which non-curative protection acts (‘durability’). In practice, this duration would also depend on the risk of exogenous reinfection in a given context [38], a factor accounted for independently in our model through the range of country settings that we examined (Table 1).

While none of these parameters is directly measurable using current assays, the purpose of delineating them is to accommodate a range of mechanisms in simulating future preventive treatment; we developed a methodology for translating any combination of these mechanisms into the efficacy that would be observed in clinical trials. In brief, we used a reduced form of the model equations to simulate a cohort of household contacts and PLHIV, in the absence of transmission and assuming full regimen adherence (the latter to simulate trial conditions). For a given set of mechanistic parameters, we simulated the incidence that would be observed in this cohort with and without preventive treatment at 2 years post-regimen completion and thus calculated the simulated efficacy (further details in Additional file 5). In our primary analysis, we thus focused on efficacy as a predictor for incidence impact, while treating the above mechanistic parameters as ‘hidden’ properties, to accommodate a range of possible scenarios for mechanisms of protection.

Second, we incorporated ‘ease-of-adherence’ to capture the ability of patients to complete the regimen under real-world programmatic conditions. Ease-of-adherence is influenced by a range of factors including pill burden, frequency of dosage and safety and tolerability. Regimen duration is also important: in practice, it is expected that shorter regimens would have higher completion rates, a key rationale for the development of recent 3- and 1-month regimens [7, 8]. However, given a lack of systematic evidence quantifying the relationship between regimen duration and completion rates in *programmatic* conditions, for simplicity, we modelled ease-of-adherence and regimen duration as playing separate roles.

Finally, we incorporated ‘forgiveness’ to capture the implications of regimen non-completion. We assumed that patients completing less than half of the regimen would not experience any preventive benefits. Amongst patients completing more than half of the regimen before interrupting, we defined ‘forgiveness’ as the proportion that would nonetheless have the same outcomes as those completing the regimen. This construction (subject to sensitivity analysis) serves to bar artificial scenarios in which patients who interrupt immediately after treatment initiation might still benefit from forgiveness.

### Assessing the role of each regimen property on epidemiologic impact

We drew 1000 realisations for possible future regimens, spanning the interval between minimal and optimal scenarios listed in Table 2. In particular, we first drew 10^4^ latin hypercube samples for all regimen properties listed in Table 2, including mechanism parameters, and excluding efficacy in this first step. With each such sample representing a different regimen, we next determined the efficacy of each regimen using the cohort-modelling approach mentioned above and described in more detail in Additional file 5. We selected, at random, 1000 regimens with efficacy between 70 and 100% (in agreement with Table 2).

Concatenating these regimen samples with the 1000 parameter samples obtained from model calibration, we then projected the cumulative incidence averted by future preventive treatment between 2021 and 2035, when scaled up over the next 3 years, to cover all PLHIV and household contacts of notified cases (consistent with WHO guidelines). We evaluated ‘epidemiological impact’ as the cumulative incidence averted, relative to a ‘status quo’ comparator where current coverage of 6H, and current TB services, were assumed to continue indefinitely. We finally evaluated the partial rank correlation coefficient (PRCC) between each regimen property and the epidemiological impact. Thus, properties showing greatest values of PRCC are most strongly associated with reduction in TB incidence and would afford higher priority in assessment of any future regimens. We repeated this analysis for each of the 4 countries listed in Table 1.

### Secondary and sensitivity analyses

In the primary analysis described above, we translated mechanism parameters to efficacy because only the latter is observable. However, understanding the role of underlying mechanisms can also be helpful for regimen development. We therefore conducted secondary analysis to assess the correlation between the three mechanism parameters (cure, suppression and waning) and efficacy.

We also performed the following sensitivity analyses: (i) whereas in the main analysis, we assumed ‘forgiveness’ would only apply for individuals completing at least 50% of the regimen before interrupting, we repeated the analysis when assuming this threshold to be at 25% and 75%. (ii) Model projections originate from the range of parameters listed in Table 2, which in turn were informed by expert consensus. To test model sensitivity to these ranges, we repeated the analysis with all parameter ranges widened by a twofold factor, relative to the midpoints of the ranges (with upper limits capped at 100% for all percentage terms). (iii) Finally, while the main analysis above was based on a ‘status quo’ comparator assuming continued levels of current coverage of 6H, we repeated this analysis using an alternative comparator of 6H scale-up. We assumed that this expansion would happen in the same manner as assumed for future preventive treatment regimens: that is, being scaled up in a linear way over the next 3 years, to cover all PLHIV and household contacts of TB cases, and with this coverage being maintained indefinitely thereafter.

## Results

Additional file 4: Figs. S3 to S6 show the results of model calibration for each of the four countries in this analysis. Given these model calibrations, Additional file 6: Fig. S11 and Table S6 illustrate the epidemiological impact of both minimal and optimal regimens, when deployed amongst all PLHIV and household contacts, in the four countries. Between 2020 and 2035, a regimen meeting minimal criteria would have a range of impacts in different settings, ranging from 4.2% (95% CrI 2.6–5.1) reduction in cumulative incidence in Brazil to 18% (95% CrI 14–21.3) reduction in South Africa, relative to a status quo comparator. A fully optimal regimen would increase this impact to 10.3% (95% CrI intervals 7–12) in Brazil, and 44.8% (95% CrI intervals 40.3–49) in South Africa, again relative to a status quo comparator. Table S6 lists these impacts by country for both types of regimens and when stratified by PLHIV and household contacts.

**Fig. 2:**
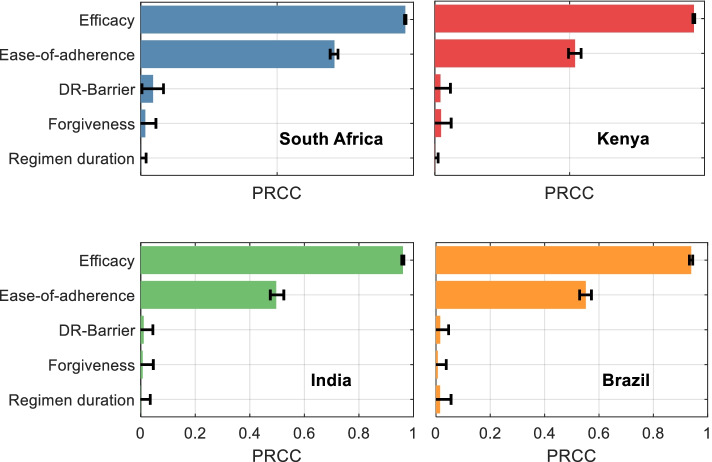
The influence of different regimen properties on potential incidence reductions from a future preventive treatment regimen. Shown are partial rank correlation coefficients (PRCCs) between each regimen property listed in Table 2, and the percent cases averted between 2020 and 2035, of a regimen that is rolled out to cover all PLHIV on ART, as well as all household contacts of notified cases. Larger bars indicate regimen properties having greater influence on incidence reductions; error bars show 95% uncertainty intervals, estimated by bootstrapping. See Fig. S9 for scatter plots underlying these correlations

Next, to identify the regimen properties that are most influential in achieving this impact, Fig. 2 shows partial rank correlation coefficients (PRCCs) of incidence reductions against each of the five modelled regimen properties. In all countries, regimen efficacy (as would be measured at 2-year follow-up under trial conditions) is the single most important predictor of incidence reduction. However, ease-of-adherence also plays an important secondary role in each country. Remaining properties, i.e., forgiveness to non-completion, barrier to developing drug resistance, and regimen duration play only minor roles in regimen impact. Regimen duration is only slightly more impactful in Brazil. Additional file 5: Fig. S9 shows the scatter plots of each property against epidemiological impact, illustrating the univariate associations underlying these PRCC estimates. Additionally, while Fig. 2 shows PRCC estimates relative to a status quo comparator (indefinite continuation of current coverage of 6H, see Additional file 7: Table S7), these model findings remain unchanged when assuming an alternative comparator of 6H scale-up, to the same levels being assumed for future regimens (Additional file 7: Fig. S12).

**Fig. 3:**
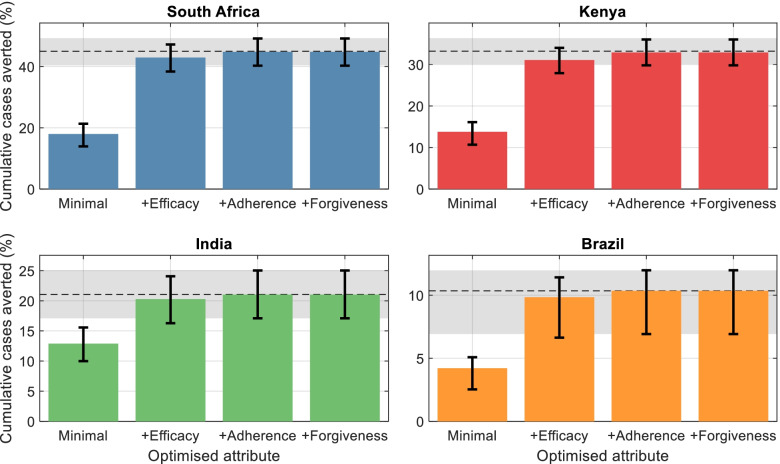
Contribution of regimen properties to epidemiological impact. While Fig. 2 shows regimen properties in order of decreasing influence on incidence reductions, this figure shows the quantitative effect of each on impact. In each panel, the leftmost bar shows the impact (cumulative cases averted) of a PT regimen with properties fulfilling only minimal criteria in Table 2. The remaining bars show the impact of successively optimising each single regimen property in turn, starting with the most influential properties shown in Fig. 2. For clarity, only the top four most influential properties are shown. The horizontal dashed line shows the impact arising from a fully optimised regimen, i.e., with all 5 properties assuming optimal values in Table 2). Error bars, and the gray shaded area, show 95% Bayesian credible intervals on respective estimates

Figure 3 shows the incremental role of each regimen property in incidence-reducing impact. Starting from a regimen satisfying only minimal criteria (left-hand bars), the figure illustrates how impact increases with each regimen property being optimised in turn, beginning with the most influential. For example, in South Africa, optimising efficacy leads to an increase of 25 percentage points in impact, relative to a minimal regimen; additionally, optimising adherence leads to a further increase of 2 percentage points in impact. In practice, given the progress that has already occurred in developing shorter, simpler preventive therapy regimens [8, 39], it seems likely that ease-of-adherence would see further improvements before regimens with improved efficacy emerge. Accordingly, Additional file 7: Fig. S13 in the supporting information shows the incremental role of each regimen property under a scenario where ease-of-adherence is optimised first. The figure illustrates how important gains could be achieved in impact, through this regimen attribute alone.

**Fig. 4:**
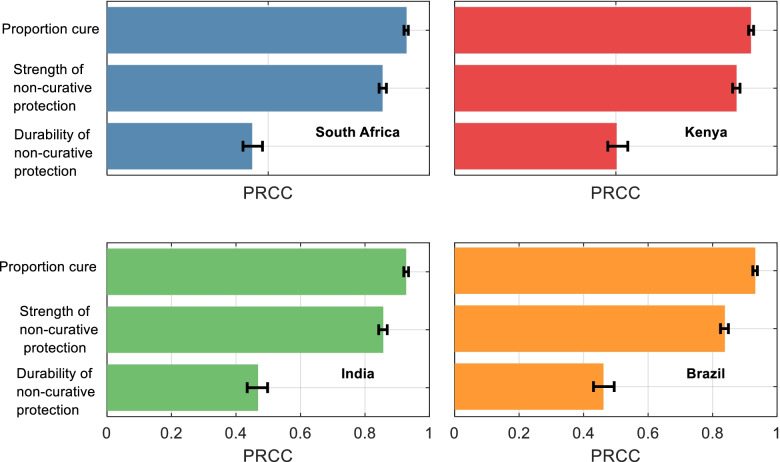
The influence of ‘mechanistic’ regimen parameters on regimen efficacy. As described in the main text, we define ‘efficacy’ as the incidence reductions that would result under trial conditions, over 2 years of follow-up. However, efficacy in the model accommodates a range of scenarios for the relative role of different, underlying mechanisms of protection. Shown are estimates for the strength of association between each mechanistic parameter, and efficacy (incidence reductions) that would be measured under trial conditions, upon 2-year follow-up. As in Fig. 2, larger bars indicate those parameters that are more influential for efficacy, and error bars show 95% uncertainty intervals, estimated through bootstrapping. See Additional file 5: Fig. S10 for further analysis on how parameters interact to yield efficacy

These results present efficacy as a key property of a regimen, based on ‘hidden’ mechanisms governing pharmacokinetic and pharmacodynamic actions. Figure 4 presents additional analyses for the influence of each of these mechanisms on efficacy, illustrating that, in all countries modelled, the proportion cured and the relative strength of non-curative protection play leading roles in determining efficacy: the post-regimen durability of non-curative protection plays only a secondary role. Thus, even regimens that do not fully cure infection can meet the criteria for regimen efficacy, as long as their non-curative protection is sufficiently strong. Additional file 5: Fig. S10 shows additional analysis for two-way parameter combinations in the example of Kenya, highlighting that a high proportion cure can compensate for a low strength of non-curative protection, and vice versa, but the highest-efficacy regimens are only possible with high values for both parameters. Again, in this analysis, the durability of non-curative protection only plays a secondary role.

Finally, Additional file 7: Figs. S14–S18 present further sensitivity analyses. While we have so far assumed that forgiveness to non-completion applies only to those completing at least half of the regimen, Additional file 7: Fig. S14 and Table S9 show results when assuming alternative thresholds of 25% and 75%, illustrating results that remain qualitatively similar to those illustrated in Fig. 2. Another assumption in the main analysis is that a future regimen would be half as effective against rifampicin-resistant infection as against rifampicin-sensitive infection. Additional file 7: Table S10 shows results under alternative assumptions of 25% and 75%, again showing qualitatively similar results to Fig. 2 on the relative importance of the various regimen properties. Additional file 7: Fig. S15 and Table S11 show additional sensitivity analysis when adopting a broader range of regimen properties than in Table 2, again illustrating qualitatively similar findings to those shown in Fig. 2. Additionally, we examined the extent to which the apparent low importance of forgiveness in Fig. 2 is driven by model assumptions. We aimed to adjust the model parameters in order to increase the importance of forgiveness as a determinant of overall impact, finding that two adjustments were needed: (i) to reduce the proportion of individuals completing the regimen (ease-of-adherence) and (ii) to reduce the assumed minimum amount of the regimen that needs to be taken, in order for forgiveness to apply. Additional file 7: Fig. S16 shows illustrative results where these parameters are reduced to 40 and 5%, respectively, showing the increased importance of forgiveness as a result. However, with published findings for 3HP showing completion rates of over 80% in practice [40], these parameter ranges are arguably too pessimistic for future regimens. Finally, we assumed that there is some protective effect during the course of preventive treatment, raising the possibility that longer regimens may contribute to improved effectiveness. However, Additional file 7: Fig. S17 illustrates that any such effect is insignificant relative to parametric uncertainty.

## Discussion

In prioritising amongst desired attributes for future TB preventive treatment regimens, assessment of their potential epidemiological impact is a key consideration. Our results illustrate that efficacy—as would be measured over a 2-year follow-up in trial conditions—is the most important predictor of incidence reduction, with an important role also being played by ease-of-adherence (Fig. 2). Ease-of-adherence has already been reported by patients as being an important regimen property [41, 42], and our work complements this patient-centred perspective by highlighting the epidemiological importance of this regimen property. In practice, optimising both efficacy and ease-of-adherence might not be achieved in a single step, but through a series of improvements in both; indeed, recent developments have shown important progress in promoting adherence through shorter, simpler regimens [7, 8]. In future, our results suggest that even interim progress in optimising either of these regimen properties would have important implications for incidence reduction (Fig. 3).

Our results have implications for data gathering for future candidate regimens. First, while efficacy data are already a key focus of clinical trials, ease-of-adherence would require additional data collection on regimen completion rates that are achievable under programmatic conditions, and on how these completion rates might feasibly be optimised through effective implementation approaches. Second, for simplicity, we have modelled regimen duration and ease-of-adherence as separate properties, while recognising that in practice, shorter durations are likely to facilitate regimen completion. In future, data comparing regimen completion rates under programmatic conditions will be invaluable in extending the model to better capture associations between duration and ease of adherence. Third, multi-year follow-up in programmatic conditions (similar to those conducted in trial conditions) would be required to evaluate additional characteristics such as forgiveness to non-completion. Overall, further integrating evaluation under programmatic conditions into the clinical development pathway of future candidate regimens, with a view to informing public health recommendations, would be an invaluable addition to their holistic assessment [43].

Notably, although the priority regimen attributes are consistent across countries (Fig. 2), the magnitude of epidemiological impact varies widely (Additional file 6: Fig. S11) with, for example, optimal regimens reducing cumulative incidence by 45% in South Africa between 2020 and 2035, but only by 10% in Brazil. A key reason for these differences is the extent to which the TB epidemic is driven by recent transmission vs the reactivation of remote infection. In South Africa, where annual incidence exceeds 500 per 100,000, model calibrations suggest that recent infection accounts for 75% of incidence and reactivation of remote infection for 16% (with the remainder arising from relapse). By contrast in Brazil, annual incidence rates are less than a tenth of those in South Africa, and rates of HIV/TB coinfection are substantially lower than in South Africa. Model calibrations suggest that In Brazil, recent and remote infection account respectively for 53% and 23% of annual incidence. Preventive therapy would be expected to have a stronger transmission impact in settings such as South Africa where recent transmission plays a dominant role, because its indirect effect is greater in such settings: that is, the number of onward transmissions that are averted by preventing one case of incident TB.

While our analysis is relevant for global recommendations, it does not address subnational variations. A prominent example is in South African gold mines, where preventive treatment was found to have only a limited durability of post-regimen protection [38], hindered by exceptionally intense, local transmission, and the high prevalence of silicosis in the study population, an important risk factor for developing TB. Locally tailored preventive strategies may be necessary in such circumstances, for example using longer-duration, or repeated, preventive treatment [44]. As described in the final target product profiles [9], repeat treatment would be facilitated by shorter and safe regimens.

Our selection of four countries addresses a range of contexts for TB epidemiology but does not address countries with a high burden of rifampicin-resistant TB, such as those in Central and Eastern Europe, and countries of the former Soviet Union. A challenge with preventive treatment of rifampicin-resistant infection is that assays for TB infection do not provide information about drug resistance status; current WHO guidelines recommend the use of fluoroquinolone-based regimens amongst contacts of TB patients known to have drug resistance [23]. Future preventive treatment regimens that are rifampicin or rifapentine sparing could be invaluable in allowing the use of a single regimen regardless of potential rifampicin resistance—our supplementary analysis (Additional file 7: Fig. S18) suggests that, for such regimens in future, priority regimen properties would be consistent with those presented in Fig. 2.

As with any modelling approach, we have had to incorporate a series of simplifications. Our model does not include age structure, motivated partly by model parsimony, and partly by the fact that current preventive treatment guidelines do not distinguish by age, whether amongst PLHIV or household contacts [4]. However, this simplification ignores some pronounced age-specific features of TB natural history, for example with (i) children having a higher risk of progressing to active disease than adults following exposure to TB, and (ii) upon developing disease, children being more likely to develop extrapulmonary or disseminated disease [45, 46], and thus also being less infectious than adult disease. Our current approach ignores these variations, essentially modelling average rates of progression and infectivity. In so doing, our model also does not address the potential implications of demographic change over the next few decades, in countries such as India [47]. Future analysis refining this work should aim to address these factors. We have made simplifying assumptions for household composition, including that the average household size for TB patients is the same as the national average, and likewise for the prevalence of HIV in these households. In practice, to the extent that TB burden and household size are both linked with socioeconomic status, household sizes may be larger for TB patients than the national average: thus, our model is likely to be conservative with respect to the impact of preventive therapy in this risk group. Amongst other simplifications, in modelling forgiveness, we have assumed a dichotomy in which forgiveness only applies to patients completing a certain threshold proportion of the regimen. In practice, it is likely that there is a more continuous relationship between forgiveness and the proportion of the regimen completed, although our sensitivity analysis illustrates that our qualitative results are not substantially altered by the choice of threshold (Additional file 7: Fig. S14). Moreover, we have focused here on forgiveness to non-completion of the regimen. Recent analysis of trial data for the treatment of active TB suggests that missed doses—during the course of the regimen—are an important predictor of post-treatment outcomes [48], and similar challenges may apply to preventive treatment as well. Distinguishing these different types of ‘forgiveness’ is beyond the scope of our current work, but an important area for future analysis. Finally, our work assumes hypothetical scenarios where future regimens are scaled up to cover all eligible populations. How such coverage will be achieved is a critical question for implementation research, especially given current, low levels of coverage amongst adult household contacts. The feasibility of increasing coverage may well depend on future regimen characteristics, for example with shorter, simpler regimens posing less of an implementation challenge for TB programmes, than daily treatment for 6 months.

With prevention taking on increasing importance in the TB response, as advocated in the WHO End TB Strategy [49], and while we await new, more efficacious and safe TB vaccines, future approaches to preventive treatment will play a critical role in global efforts to end TB. As innovations continue in the development of new preventive treatment regimens, crystallising priorities for these future preventive tools carries important implications for their evaluation and impact in the coming years and beyond.

## Conclusions

As preventive therapy regimens continue to undergo development, there is a need to understand what regimen properties will be most important for epidemiological impact. To our knowledge, ours is the first study to address this question. Using regimen properties distilled from the WHO target product profiles for future TB regimens, we examined which of these properties would have the greatest influence on epidemiological impact in each of the four focal countries. For epidemiological impact, the most important characteristics for future preventive therapy regimens are consistent across a wide range of country settings: regimen efficacy is the strongest predictor, followed by ease-of-adherence. Other regimen properties, such as barrier to developing drug resistance, play only minor roles in the projected epidemiological impact. Complementing previous, patient-centred studies of regimen preference, our analysis supports the importance of shorter, simpler regimens that facilitate ease-of-adherence, while also emphasising the need for improved efficacy in future regimen development.

## Supplementary Information

**Additional file 1. **Model technical details. **Fig. S1.** Further model structure schematic. **Table S1.** Model symbols. Model equations 1-25.

**Additional file 2. **Model parameters. **Table S2.** List of model parameters.

**Additional file 3. **Methods for simulating household TPT. **Fig. S2.** TPT cohort model. **Table S3.** Parameters for household TPT.

**Additional file 4. **Details of model calibration. **Table S4.** Calibration targets. **Figs. S3-S6.** Calibration results by country. **Fig. S7.** MCMC diagnostics.

**Additional file 5. **Translating mechanisms of protection to efficacy. **Table S5.** Mechanistic parameters of efficacy. **Fig. S8.** Simulation of cohort efficacy. **Fig. S9.** Scatter plots of regimen properties. **Fig. S10.** Correlations between mechanistic parameters.

**Additional file 6. **Epidemiological impact of TPT by country. **Fig. S11.** Incidence curves under different TPT scenarios. **Table S6.** Summary of epidemiological impact by country.

**Additional file 7. **Further sensitivity analysis: **Tables S7-S8** and **Fig. S12.** Results with 6H scale-up as alternative comparator. **Fig. S13.** Alternative optimisation order of regimen properties. **Table S9** and **Fig. S14.** Sensitivity analysis on forgiveness thresholds. **Table S10.** Sensitivity analysis on efficacy of regimen on Rif resistant TB. **Fig. S15** and **Table S11.** Widened ranges for regimen properties. **Fig. S16.** PRCCs assuming lower ease of adherence. **Fig. S17.** Effect of regimen duration on epidemiological impact. **Fig. S18.** PRCCs for a Rifampicin-sparing regimen.

## Data Availability

All used in the study for model parameterisation is publicly available (see Additional files 2 and 4) and data for model calibration is available from the *World Health Organization. Global Tuberculosis Report 2020.*
