# Supplementary material for: Prioritising attributes for tuberculosis preventive treatment regimens: a modelling analysis
Source: BMC Med. 2022 May 18;20:182. doi: 10.1186/s12916-022-02378-1 (PMC9115962; doi:10.1186/s12916-022-02378-1)
Supplement: Supplementary file 1 — Additional file 1. Model technical details. Fig. S1. Further model structure schematic. Table S1. Model symbols. Model equations 1-25. [file 12916_2022_2378_MOESM1_ESM.pdf]

Additional file No. 1: Model technical details

Fig. S1 provides further schematic illustration of the model structure, supplementary to Figure 1 in the main text. Table S1 lists the symbols used for model compartments; the subsequent text lists the model equations, and Table S2 lists all model parameters.

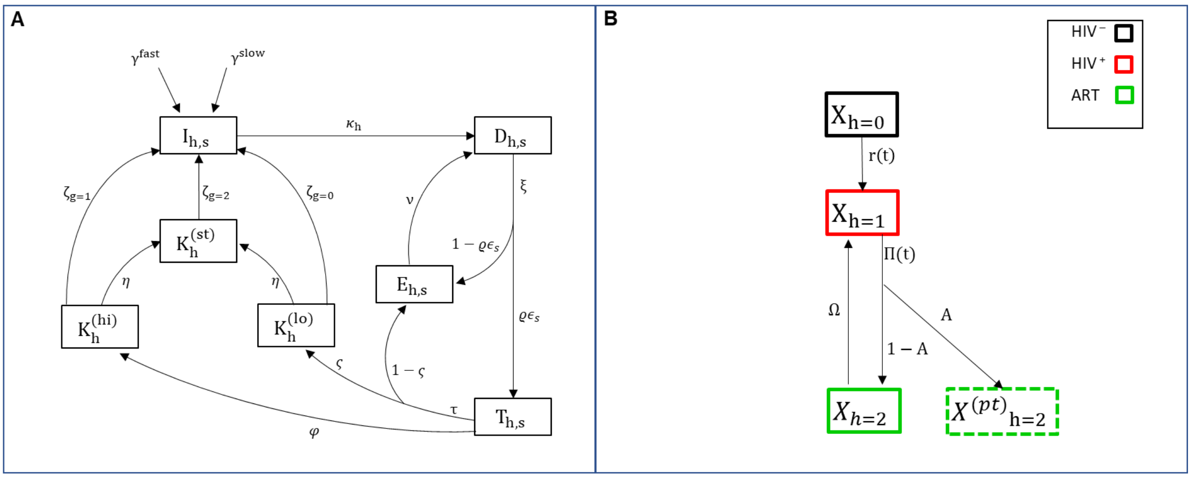

Figure S1 Further schematic illustration of the model structure. *Figure 1 in the main text shows model structure relevant to the preventive treatment cascade. Here, panel (A) elaborates on the care cascade for active TB (encapsulated in the box labelled 'TB care' in Figure 1), while panel (B) illustrates how model compartments are further stratified by HIV status. In the latter, HIV-negative individuals acquire HIV at a time-varying rate h(t) (transitions from black to red boxes). The schematic shows transitions for ART initiation (transitions from black to red boxes), as well as the uptake of preventive treatment among those initiating ART (transitions to green boxes). For rates of ART initiation, we drew from UNAIDS estimations for each country. See table S1 for definitions of subscripts and model stages, and Additional file 2 table S2 for parameter definitions and values.*

Symbol	Meaning
U	TB Uninfected
$L^{(fast)}$	TB infected, 'fast' reactivation
$L^{(slow)}$	TB infected, 'slow' reactivation
I	Active TB, prior to care-seeking
D	Active TB, awaiting diagnosis
E	Between care-seeking episodes (following missed diagnosis or initial loss to follow-up)
$T;\ Fl$	First Line TB treatment
$T;\ Sl$	Second line TB treatment
$K^{(hi)}$	Post-treatment recovery, high risk of relapse
$K^{(lo)}$	Post-treatment recovery, low risk of relapse
$K^{(st)}$	Stabilised risk of relapse
$P^{(1)}$	Undergoing preventive treatment, first half of regimen
$P^{(2)}$	Undergoing preventive treatment, second half of regimen
S	Cured of TB infection by preventive therapy
Q	Completed preventive treatment and undergoing non-curative, post-regimen protection
$R^{(fast)}$	Back to fast progression status after lapse of post regimen protection
$R^{(slow)}$	Back to slow progression status after lapse of post regimen protection
**Subscripts**	
i	State of TB infection (LTBI) state, 0=fast 1=slow
h	HIV status: 0=HIV negative; 1= HIV + undetected ; 2= HIV+ on ART
s	Strain: 0= DS ; 1= MDR;

Table S1 ***Definition of model compartments****. These symbols are used in Figure S1, and the model equations in the subsequent sections.*

The following ordinary differential equations correspond to the model compartments described in Fig1 in main text and FigS1.

Uninfected

$$\frac{dU_h(t)}{dt} = \begin{cases} Z_h - U_h(t)\lambda_s(t) - U_h(t)\upsilon\omega + W_h^{(1)}(t) - U_h(t)\mu_h & for\ h = 0 \\ -U_h(t)\lambda_s(t) - U_h(t)\upsilon\omega + W_h^{1}(t) - U_h(t)\mu_h & for\ h > 0 \end{cases}, \qquad (1)$$

Latent fast

$$\frac{dL_{h,s}^{(f)}(t)}{dt} = \lambda(t)\left[U_h(t) + \iota\left(L_{h,s}^{(s)} + K_h^{(lo)}(t) + K_h^{(hi)}(t) + K_h(t)\right)\right] + W_k^{(2)}(t) - L_{h,s}^{(f)}(t)\left(\mu_h + \chi\psi\omega + \gamma^{(f)} + \delta\right), \qquad (2)$$

Latent slow

$$\frac{dL_{h,s}^{(s)}(t)}{dt} = L_{h,s}^{(f)}\delta + W_h^{(3)}(t) - L_{h,s}^{(s)}(t)\left(\mu_h + \psi\omega + \gamma^{(s)} + \lambda(t)\iota\right), \qquad (3)$$

First half of PT administration (uninfected)

$$\frac{dP1_h(t)}{dt} = U_h(t)\upsilon\omega + W_h^{(4)}(t) - P1_h(t)(\mu_h + d + m), \qquad (4)$$

First half of PT administration (from latent fast)

$$\frac{dP1_{h,s}^{(f)}(t)}{dt} = \lambda(t)\left[P1_h(t) + P1_{h,s}^{(s)}\iota\right] + L_{h,s}^{(f)}\chi\psi\omega + W_h^{(5)}(t) - P1_{h,s}^{(f)}(t)\left(\mu_h + d + m + c + e\gamma^{(f)} + \delta\right), \qquad (5)$$

First half of PT administration (from latent slow)

$$\frac{dP1_{h,s}^{(s)}(t)}{dt} = L_{h,s}^{(s)}\psi\omega + P1_{h,s}^{(f)}(t)\delta + W_h^{(6)}(t) - P1_{h,s}^{(f)}(t)\left(\mu_h + d + m + c + e\gamma^{(s)}\right), \qquad (6)$$

Latent infection cured by PT regimen

$$\frac{dS_h(t)}{dt} = c\left(P1_{h,s}^{(f)}(t) + P2_{h,s}^{(f)}(t) + P1_{h,s}^{(s)}(t) + P2_{h,s}^{(s)}(t)\right) + W_h^{(7)}(t) - S_h(t)(\mu_h + \lambda(t)\iota), \qquad (7)$$

Second half of PT administration (uninfected)

$$\frac{dP2_h(t)}{dt} = P1_h(t)m + W_h^{(8)}(t) - P2_h(t)(\mu_h + d + m), \qquad (8)$$

Second half of PT administration (from latent fast)

$$\frac{dP2_{h,s}^{(f)}(t)}{dt} = \lambda(t)\left[P2_h(t) + P2_{h,s}^{(s)}\iota\right] + P1_{h,s}^{(f)}(t)m + W_h^{(9)}(t) - P2_{h,s}^{(f)}(t)\left(\mu_h + d + m + c + e\gamma^{(f)} + \delta\right), \qquad (9)$$

Second half of PT administration (from latent slow)

$$\frac{dP2_{h,s}^{(s)}(t)}{dt} = P1_{h,s}^{(s)}(t)m + P2_{h,s}^{(f)}(t)\delta + W_h^{(10)}(t) - P2_{h,s}^{(s)}(t)\left(\mu_h + d + m + c + e\gamma^{(s)}\right), \qquad (10)$$

Post PT regimen (uninfected)

$$\frac{dQ_h(t)}{dt} = P2_h(t)m + d(P1_h(t) + P2_h(t)) + W_h^{(11)}(t) - Q_h(t)\left(\mu_h + \lambda(t)\right), \qquad (11)$$

Post PT regimen (from latent fast)

$$\frac{dQ_{h,s}^{(f)}(t)}{dt} = \begin{cases} P2_{h,s}^{(f)}(t)m + P2_{h,s}^{(f)}(t)dfb + W_h^{(12)}(t) - Q_{h,s}^{(f)}(t)\left(\mu_h + g + e\gamma^{(f)} + \delta\right), for\ s = 0 \\ P2_{h,s}^{(f)}(t)m + P2_{h,s}^{(f)}(t)df(1-b) + W_h^{(12)}(t) - Q_{h,s}^{(f)}(t)\left(\mu_h + g + e\gamma^{(f)} + \delta\right), for\ s = 1 \end{cases} \qquad (12)$$

Post PT regimen (from latent slow)

$$\frac{dQ_{h,s}^{(s)}(t)}{dt} = \begin{cases} P2_{h,s}^{(s)}(t)m + P2_{h,s}^{(s)}(t)dfb + Q_{h,s}^{(f)}(t)\delta + W_h^{(13)}(t) - Q_{h,s}^{(s)}(t)\left(\mu_h + g + e\gamma^{(s)} + \lambda(t)\iota\right), for\ s = 0 \\ P2_{h,s}^{(s)}(t)m + P2_{h,s}^{(s)}(t)df(1-b) + Q_{h,s}^{(f)}(t)\delta + W_h^{(13)}(t) - Q_{h,s}^{(s)}(t)\left(\mu_h + g + e\gamma^{(s)} + \lambda(t)\iota\right), for\ s = 1 \end{cases} \quad (13)$$

Back to fast progression track after PT protection

$$\frac{dR_{h,s}^{(f)}(t)}{dt} = \begin{cases} P1_{h,s}^{(f)}(t)db + P2_{h,s}^{(f)}(t)d(1-f)b + Q_{h,s}^{(f)}(t)g + W_h^{(14)}(t) - R_{h,s}^{(f)}(t)\left(\mu_h + \gamma^{(f)} + \delta\right), for\ s = 0 \\ P1_{h,s}^{(f)}(t)db + P2_{h,s}^{(f)}(t)d(1-f)(1-b) + Q_{h,s}^{(f)}(t)g + W_h^{(14)}(t) - R_{h,s}^{(f)}(t)\left(\mu_h + \gamma^{(f)} + \delta\right), for\ s = 1 \end{cases} \quad (14)$$

Back to fast progression track after PT protection

$$\frac{dR_{h,s}^{(s)}(t)}{dt} = \begin{cases} P1_{h,s}^{(s)}(t)db + P2_{h,s}^{(s)}(t)d(1-f)b + Q_{h,s}^{(s)}(t)g + R_{h,s}^{(f)}(t)\delta + W_h^{(15)}(t) - R_{h,s}^{(s)}(t)\left(\mu_h + \gamma^{(s)}\right), for\ s = 0 \\ P1_{h,s}^{(s)}(t)db + P2_{h,s}^{(s)}(t)d(1-f)(1-b) + Q_{h,s}^{(s)}(t)g + R_{h,s}^{(f)}(t)\delta + W_h^{(15)}(t) - R_{h,s}^{(s)}(t)\left(\mu_h + \gamma^{(s)}\right), for\ s = 1 \end{cases} \quad (15)$$

Active TB

$$\frac{dI_{h,s}(t)}{dt} = \gamma^{(f)}\left(L_{h,s}^{(f)}(t) + R_{h,s}^{(f)}(t)\right) + e\gamma^{(f)}\left(P1_{h,s}^{(f)}(t) + P2_{h,s}^{(f)}(t) + Q_{h,s}^{(f)}(t)\right) + \gamma^{(s)}\left(L_{h,s}^{(s)}(t) + R_{h,s}^{(s)}(t)\right) + e\gamma^{(s)}\left(P1_{h,s}^{(s)}(t) + P2_{h,s}^{(s)}(t) + Q_{h,s}^{(s)}(t)\right) + K_h^{(lo)}(t)\zeta_0 + K_h^{(hi)}(t)\zeta_1 + K_h^{(st)}(t)\zeta_2 + W_h^{(16)}(t) - I_{h,s}(t)\left(\mu_h^{(tb)} + \mu_h + \kappa_h\right), \quad (16)$$

Initial presentation to care & awaiting for diagnosis

$$\frac{dD_{h,s}(t)}{dt} = I_{h,s}(t)\kappa_h + E_{h,s}(t)\nu + W_h^{(17)}(t) - D_{h,s}(t)\left(\mu_h^{(tb)} + \mu_h + \xi\right), \quad (17)$$

Missed Diagnosis and initial loss to follow-up

$$\frac{dE_{h,s}(t)}{dt} = D_{h,s}(t)\xi(1 - \varrho\epsilon_s) + Fl_{h,s}(t)\tau^{(fl)}\left(1 - \varsigma^{(\mathrm{fl})}\right) + Sl_{h,s}(t)\tau^{(sl)}\left(1 - \varsigma^{(\mathrm{sl})}\right) + W_h^{(18)}(t) - E_{h,s}(t)\left(\mu_h^{(tb)} + \mu_h + \nu\right), \quad (18)$$

First line TB treatment

$$\frac{dFl_{h,s}(t)}{dt} = D_{h,s}(t)\xi\varrho\epsilon_s + W_h^{(19)}(t) - Fl_{h,s}(t)\left(\mu_h^{(fl)} + \mu_h + \tau^{(fl)} + \varphi^{(fl)}\right), \quad (19)$$

Second line TB treatment

$$\frac{dSl_{h,s}(t)}{dt} = D_{h,s}(t)\xi\varrho\epsilon_s + W_h^{(20)}(t) - Sl_{h,s}(t)\left(\mu_h^{(sl)} + \mu_h + \tau^{(sl)} + \varphi^{(sl)}\right), \quad (20)$$

Recovery after curative treatment (low relapse risk)

$$\frac{dK_h^{(lo)}(t)}{dt} = Fl_{h,s}(t)\tau^{(fl)}\varsigma^{(\mathrm{fl})} + W_h^{(21)}(t) - K_h^{(lo)}(t)(\mu_h + \eta + \zeta_0), \quad (21)$$

Recovery after curative treatment (high relapse risk)

$$\frac{dK_h^{(hi)}(t)}{dt} = Fl_{h,s}(t)\varphi^{(fl)} + Sl_{h,s}(t)\varphi^{(sl)} + W_h^{(22)}(t) - K_h^{(hi)}(t)(\mu_h + \eta + \zeta_1), \quad (22)$$

Recovery after curative treatment (stable relapse risk)

$$\frac{dK_h^{(hi)}(t)}{dt} = \eta\left(K_h^{(lo)}(t) + K_h^{(hi)}(t)\right) + W_h^{(23)}(t) - K_h^{(st)}(t)(\mu_h + \zeta_2), \quad (23)$$

In equations 1 to 23 the terms in $W_h^l(t)$ represent transitions between HIV stages which, for clarity, are listed separately below.

Force of Infection

$$\lambda_s(t) = \begin{cases} \frac{\beta_{ds}\{\sum_{h=1}^{2}[I_{h,s}(t)+D_{h,s}(t)+E_{h,s}(t)]\}+\beta_{ds}\alpha\{\sum_{h=1}^{2}[I_{h,s}(t)+D_{h,s}(t)+E_{h,s}(t)]\}}{N(t)}, & for\ s=0 \\ \frac{\beta_{dr}\{\sum_{h=1}^{2}[I_{h,s}(t)+D_{h,s}(t)+E_{h,s}(t)]\}+\beta_{dr}\alpha\{\sum_{h=1}^{2}[I_{h,s}(t)+D_{h,s}(t)+E_{h,s}(t)]\}}{N(t)}, & for\ s=1 \end{cases}$$

(*24)*

where *N(t)* represents the total population at time *t.* Sub index *s* denotes the strain of TB (with *s=0* for DS and *s=1* for DR).

Transitions through HIV stages

We write $X_h^l(t)$ to represent any one of the compartments in FigS1A, with HIV transition dimension *s*, and TB dimension *l*. The following equations represent the dynamics of HIV and ART

HIV transitions

$$W_h^l = \begin{cases} -X_h^l(t)h(t) & for\ h=0 \\ X_0^l(t)h(t) - X_h^l(t)e(t)\Omega_l\Upsilon + \sum_{h=2}^{4}\left(X_h^l(t)\right)\alpha, & for\ h=1 \\ X_h^l(t)e(t)\Omega_l\Upsilon(1-f) - X_h^l(t)\Lambda(t) - X_h^l(t)\alpha, & for\ h=2 \end{cases}$$

(25)

We write super index *l* to indicate model stages in the TB transition sequence described in the equations *1 to 23* (e.g. *i = 1 for TB uninfected stage*).
