# Supplementary material for: Prioritising attributes for tuberculosis preventive treatment regimens: a modelling analysis
Source: BMC Med. 2022 May 18;20:182. doi: 10.1186/s12916-022-02378-1 (PMC9115962; doi:10.1186/s12916-022-02378-1)
Supplement: Supplementary file 2 — Additional file 2. Model parameters. Table S2. List of model parameters. [file 12916_2022_2378_MOESM2_ESM.pdf]

Additional file No. 2: Model parameters

Parameter	Symbol	Value				Source/Notes
		South Africa	Kenya	India	Brazil	
**TB natural history**						
Mean rate of transmission per DS-TB case	β_{ds}	5.1 (95% CrI 2.3 – 10.6)	8.8 (95% CrI 3.5 – 19)	11 (95% CrI 5 – 24)	11 (95% CrI 9.5 – 14)	Model estimate
Mean rate of transmission per DR-TB case	β_{dr}	4.3 (95% CrI 2 – 7.7)	7.2 (95% CrI 3 – 16)	7.5 (95% CrI 3.6 – 16)	5.7 (95% CrI 5 – 6.9)	Model estimate
TB infectiousness in $\boldsymbol{HIV^{+}}$ relative to $\boldsymbol{HIV^{-}}$	α	0.77(95% CrI 0.61 – 0.99)	0.8 (95% CrI 0.6 – 0.98)	0.8 (95% CrI 0.61 – 0.98)	0.83 (95% CrI 0.6 – 0.99)	Model estimate
Breakdown to active disease in slow progressors	γ^{slow}	0.000594				Menzies et al [10]
Breakdown to active disease in fast progressors	γ^{fast}	0.19 (95% CrI 0.07 – 0.6)	0.09 (95% CrI 0.03 – 0.24)	0.11 (95% CrI 0.05 – 0.24)	0.13 (95% CrI 0.11 – 0.15)	Model estimate
Rate of transition to the slow latent compartment	δ	0.87				Menzies et al [10]
Increased progression to TB in $\boldsymbol{HIV^{+}}$ relative to $\boldsymbol{HIV^{-}}$	ε	26				Getahun et al [12]

Relapse, per-capita hazard rates	ζ_g	g = 0; relapse following treatment completion	0.032			Driver et al [13], Thomas et al [14], Menzies et al [15]
		g = 1; relapse following treatment default	0.14			
		g = 2; relapse >2 years after treatment	0.0015			
'Stabilisation' of relapse risk following treatment	η	0.5				Based on Thomas et al: most relapse occurs in first 2 yr after treatment.
TB mortality rate	$\mu^{(tb)}$	0.16 (95% CrI 0.12 – 0.2)	0.16 (95% CrI 0.12 – 0.19)	0.16 (95% CrI 0.12 – 0.19)	0.08 (95% CrI 0.08 – 0.099)	Specified together to yield ~50% cure, ~50% mortality in average of 3 years. Tiemersma et al [16]
Spontaneous cure	θ	0.15 [0.14 –0.18]				
Relative Risk of TB mortality in HIV^+	$RR^{(h+)}$	3.3 (95% CrI 1.25-5.8)	3.7 (95% CrI 1.2-5.8)	1.6 (95% CrI 1-3.4)	2.9 (95% CrI 1.4-5)	Model estimate
Reduced susceptibility from past infection	ι	[0.25 –0.75]				Assumed range (uniform distribution)
**Health system**						
Per-capita rate of initial presentation to care	κ	2.8 (95% CrI 1 – 5.9)	1.76 (95% CrI 0.71 – 6.2)	2.4 (95% CrI 1.2 – 4.2)	5 (95% CrI 3.9 – 7)	Model estimate: corresponds to mean initial patient delay of 4.6 months (95% CrI 3.6 – 6)

Rate of Interval between care-seeking episodes (factor increase relative to initial careseeking)	ν	2				Model assumption corresponds to delay decrease of 50% between careseeking episodes
Treatment initiation delay	ξ	52				Sreeramareddy et al [17]; corresponds a mean treatment delay of 1 week
Probability of diagnosis per patient-provider interaction	o_h	h=0; 0.7 (95% CrI 0.51 – 0.94)	h=0; 0.71 (95% CrI 0.5 – 0.97)	h=0; 0.71 (95% CrI 0.5 – 0.95)	h=0; 0.9 (95% CrI 0.79 – 0.97)	Model estimate
		h=1; 0.73 (95% CrI 0.52 – 0.97)	h=1; 0.72 (95% CrI 0.51 – 0.96)	h=1; 0.81 (95% CrI 0.5 – 0.97)	h=1; 0.7 (95% CrI 0.51 – 0.96)	
Probability of rapid DST during diagnostic attempt	ϖ	71%	47%	15%	33%	WHO Country profiles
Rapid DST sensitivity	$\rho^{(xp)}$	90%				Steingart et al [18]
Smear test sensitivity	$\rho^{(sm)}$	80%				Steingart et al [18]
Treatment initiation probability	ϱ	0.72 (95% CrI 0.51 – 0.96)	0.67 (95% CrI 0.5 – 0.94)	0.74 (95% CrI 0.51 – 0.95)	0.89 (95% CrI 0.69 – 0.97)	Model estimate
Overall diagnostic probability	ϵ_s	$\epsilon_s = o_h\left(\varpi\rho^{(xp)} + (1-\varpi)\rho^{(sm)}\right), \text{for } s = 0$				Reflects the probability of diagnosis given is a DS strain

		$\epsilon_s = o_h \varpi \rho^{(xp)}$, for s = 1				Reflects the probability of diagnosis given is a DR strain
Proportion completing first line treatment	$\sigma^{(fl)}$	84.19%	87.58%	90.79%	71.02%	WHO TB programme data [19]
Proportion completing second line treatment	$\sigma^{(sl)}$	55.2%	72.0%	50.9%	61.2%	WHO TB programme data [19]
Proportion failing first line treatment	$x^{(fl)}$	0.38%	0.39%	0.93%	0.05%	WHO TB programme data [19]
Proportion failing second line treatment	$x^{(sl)}$	3.27%	1.04%	9.11%	5.31%	WHO TB programme data [19]
Proportion lost to follow-up in first line treatment	$y^{(fl)}$	8.3%	5.4%	4.5%	10.7%	WHO TB programme data [19]
Proportion lost to follow-up in second line treatment	$y^{(sl)}$	20.1%	8.3%	20.3%	20.9%	WHO TB programme data [19]
Proportion dying in first line treatment	$j^{(fl)}$	7.1%	6.6%	3.8%	7.8%	WHO TB programme data [19]
Proportion dying in second line treatment	$j^{(sl)}$	21.4%	18.7%	19.7%	11.2%	WHO TB programme data [19]
First line treatment duration	$\tau^{(fl)}$	2 y^{-1}				Corresponds to 6 month duration for standard first-line regimen. WHO guidelines [20]

Second line treatment duration	$\tau^{(sl)}$	$0.5\ y^{-1}$				Corresponds to 24 month duration for standard second-line regimen. WHO [20]
Probability of cure after first line completion	$\varsigma^{(fl)}$	$\varsigma^{(fl)} = \frac{\sigma^{(fl)}}{(\sigma^{(fl)} + x^{(fl)})}$				
Probability of cure after second line completion	$\varsigma^{(sl)}$	$\varsigma^{(sl)} = \frac{\sigma^{(sl)}}{(\sigma^{(sl)} + x^{(sl)})}$				
First line default hazard	$\varphi^{(fl)}$	$\varphi^{(fl)} = y^{(fl)}\frac{\tau^{(fl)}}{(\sigma^{(fl)} + x^{(fl)})}$				
Second line default hazard	$\varphi^{(sl)}$	$\varphi^{(sl)} = y^{(sl)}\frac{\tau^{(sl)}}{(\sigma^{(sl)} + x^{(sl)})}$				
Mortality hazard during first line treatment	$\mu^{(fl)}$	$\mu^{(sl)} = j^{(fl)}\frac{\tau^{(fl)}}{(\sigma^{(fl)} + x^{(fl)})}$				
Mortality hazard during first line treatment	$\mu^{(sl)}$	$\mu^{(sl)} = j^{(sl)}\frac{\tau^{(sl)}}{(\sigma^{(sl)} + x^{(sl)})}$				
Rate of recruitment into ART	Π	4.4 (95% CrI 3.3 – 6.8)	8 (95% CrI 5.5 – 9.8)	5.5 (95% CrI 3.3 – 9.5)	4.9 (95% CrI 3.5 – 9.1)	Model estimate
Fraction of new ART starters enrolled in PT	A	0.7 (95% CrI 0.54 – 0.82)				Model estimate
**Preventive therapy (parameters for a baseline 6 months course of isoniazid -6H)**						
PT regimen duration (months)	Γ	6				WHO LTBI treatment guidelines [4]
Rate of transition over half-course of PT	m	$m = \frac{\Gamma}{2}$				Assumption
Ease of adherence (completion)	Θ	70%				Alsdurf et al [6]
PT default hazard	d	$d = \frac{12(1-\Theta)}{\Gamma\Theta}$				

Forgiveness of non-completion	f	25%				Assumption
Waning of PT effect	g	$g = \frac{12}{60}$				To reflect an annualised rate of 60 months average protection
Suppression of reactivation effect	e	70%				Whalen et al [21]
Curative effect of PT	c	0%				Assumption
PT *Rif* resistance barrier	b	100%				Assumption: 6H regimen cannot induce Rif resistance
**Demographics**						
Birth rate	B	0.013	0.023	0.01	0.008	WHO (GHO) [21]–adjusted to yield annual population growth from 1970
Background mortality rate	μ	0.016	0.015	0.016	0.013	WHO (GHO)[21], corresponds to mean life expectancy

Table S1 ***List of model parameters****. Symbols are as used in the model equations, listed above. Numbers in brackets show 95% uncertainty intervals.*
