# Supplementary material for: Prioritising attributes for tuberculosis preventive treatment regimens: a modelling analysis
Source: BMC Med. 2022 May 18;20:182. doi: 10.1186/s12916-022-02378-1 (PMC9115962; doi:10.1186/s12916-022-02378-1)
Supplement: Supplementary file 3 — Additional file 3. Methods for simulating household TPT. Fig. S2. TPT cohort model. Table S3. Parameters for household TPT. [file 12916_2022_2378_MOESM3_ESM.pdf]

Additional file No. 3: Methods for simulating household TPT

Simulating uptake of TB preventive treatment

As an intervention we simulated full uptake of WHO guidelines for LTBI treatment guidelines, i.e. preventive treatment among 100% of PLHIV starting on ART, and among 100% of household contacts of notified TB cases.

For PLHIV, as illustrated in Additional file 1 FigS1, we assumed that amongst those initiating ART, a proportion A undergoes preventive treatment. We modelled the full implementation of WHO guidelines in this population by increasing A from its current country-specific value (obtained from WHO data) to 1, over a period of three years, and maintained at this level thereafter.

Modelling household contacts presents some challenges, as compartmental models do not lend themselves to modelling of household structure [25,26], and studies aiming to capture this structure typically employ more complex, individual-based approaches instead [27]. However, compartmental models have the advantage of simplicity and ease-of-calibration. We developed the following approach for modelling preventive treatment amongst household contacts within compartmental models.

Our approach rests on the fact that household contacts with TB infection are at greater risk of developing active disease than those infected in the general population, being more likely to have arisen from recent infection. For example, a study involving longitudinal follow up of household contacts of diagnosed TB cases showed that the TB incidence in the cohort of contacts was 8 times greater that of the general population [23].

Here FigS3 shows how this feature of close contacts could be captured within a simple compartmental representation of TB natural history, extracted from the overall model structure shown in Fig 1 in the min text. It is assumed that TB infection can be divided into an early 'high risk' stage (Lf for 'fast progressors') and a subsequent 'low risk' stage (Ls for 'slow progressors'), with individuals progressing from the first to the second if they do not develop TB disease. A recent systematic review showed how this structure is consistent with available epidemiological data from the pre-chemotherapy era [10]. Because this model does not explicitly incorporate household structure, the number in each of these compartments at a given point in time should be interpreted as representing *population-level* prevalence in each of the different states.

The vertical, dashed arrows illustrate the uptake of preventive therapy in this population, amongst household contacts of TB cases. Because TB-infected household contacts are more likely to have had recent infection than those in the general community, there would be an over-representation of individuals from the 'latent fast' ($HH^{(Lf)}$) compartment: that is, we have $\chi > 1$. If the incidence of TB amongst household contacts is (say) *k* times that of the general population, neglecting for now the effect of preventive treatment on incidence, we have that:

$$\int_{\Delta t} \gamma^{fast} HH^{(Lf\}} + \gamma^{slow} HH^{(Ls\}} dt = k \int_{\Delta t} \gamma^{fast} L_f + \gamma^{slow} L_s dt, \qquad (26)$$

where Δt is a one-year time interval. For a given *k and* ψ (in Fig.S3), it is possible to satisfy this equation by adjusting χ appropriately (in Fig.S3). It remains to determine ψ. To do so, we note that the total number of household contacts initiated on preventive therapy in a given year is:

$$\int_{\Delta t} \chi\psi Lf + \psi Ls \, dt = (hs - 1)N, \quad (27)$$

where *N* is the number of individuals with TB notified in a given year, and *hs* is the average household size (table S3). Given country-specific estimates for both of these parameters, [equation 26] therefore supplies a criterion for ψ to satisfy.

To implement this approach, we took as initial conditions the state values of the calibrated model simulated at 2020 (i.e. prior to the intervention of scaling up preventive treatment). For given values of ψ, χ, we then simulated the model forward by an interval of $\Delta t = 1$ year, assuming preventive therapy to have no efficacy. Based on the model outputs, we then used simple least-squares optimisation to determine the values of ψ, χ satisfying equations 26 and 27 simultaneously. For all subsequent years we held χ constant, while allowing ψ to vary in proportion to annual notifications.

The output of this process is to identify a value for χ, and an annual timeseries for ψ. To model the impact of preventive therapy amongst household contacts, we then simulated the model dynamics on incorporating χ, ψ, at the same time as allowing a non-zero efficacy for preventive treatment.

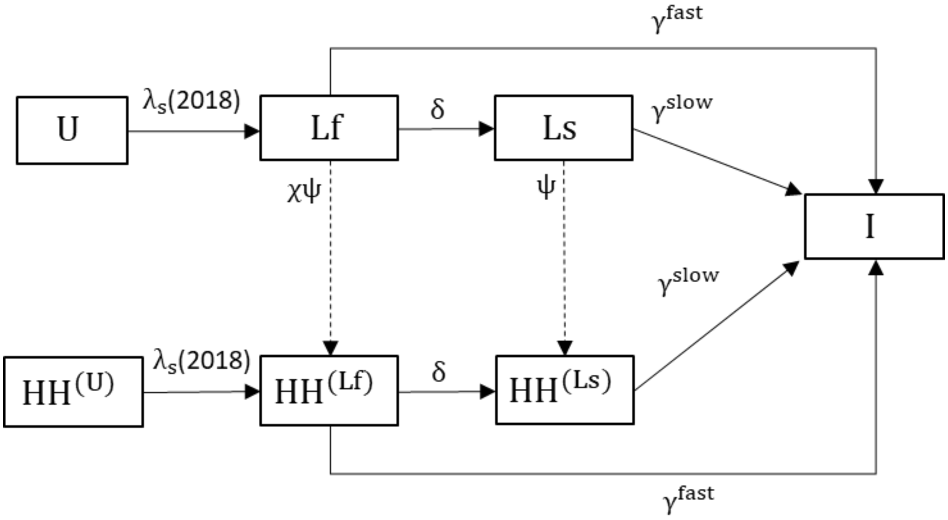

Fig S2 ***Cohort model of community and household contacts****. State variables in the upper row show untreated individuals, while those in the lower row show household contacts undergoing preventive treatment. State variables are as follows: uninfected (U); latent fast progression (Lf); latent slow progression (Ls); active disease (I); and correspondingly for the states in the lower row. Vertical, dashed arrows show the recruitment of household contacts onto preventive treatment. As described in the text, the elevated risk amongst household contacts can be modelled as a disproportionate number of individuals in the compartment HH(Lf), implemented by taking χ>1; in the model we aim to identify the parameters χ,ψ in such a way as to yield agreement with data for: (i) the TB incidence amongst infected household contacts relative to infections in the general community, and (ii) the total number of preventive treatment initiations expected in a given year, as determined by TB notifications, and average household size.*

Country	Number notified 2019*	Average Household Size+	Target of HH on PT over 4 years
Kenya	84,345	3.9	1,005,720
South Africa	209,545	3.2	2,031,392
India	2,162,323	4.8	29,408,808
Brazil	83,547	4	797,962

Table S3 ***Estimated number of household contacts in each country***

**Taken from WHO TB country profiles*
+From UN population database
