# Supplementary material for: Prioritising attributes for tuberculosis preventive treatment regimens: a modelling analysis
Source: BMC Med. 2022 May 18;20:182. doi: 10.1186/s12916-022-02378-1 (PMC9115962; doi:10.1186/s12916-022-02378-1)
Supplement: Supplementary file 4 — Additional file 4. Details of model calibration. Table S4. Calibration targets. Figs. S3-S6. Calibration results by country. Fig. S7. MCMC diagnostics. [file 12916_2022_2378_MOESM4_ESM.pdf]

Additional file No. 4: Details of model calibration

Model Calibration

Model implementation

Under a given set of parameters, we first simulated the model to equilibrium following introduction of TB into a disease-free population, with no population growth and no interventions. Then, from 1970 onwards, we increased the birth rate *b* to capture an annual population growth for each of the selected countries. We simulated the introduction of DOTS as a linear increase in the proportion of patients receiving NTP services, from 1990 to 1997. Finally simulating the model forward to 2020, we compared the model outputs for prevalence, incidence and other indicators against available data (table S4 for calibration targets).

Country	TB Incidence per 100K		MDR-TB incidence per 100K	TB Incidence per 100K (HIV+ only)	TB Mortality per 100K (HIV-)	TB Mortality per 100K (HIV+)	TB notification rate per 100K	Proportion of HIV^{+} on ART (%)*	Proportion of those on ART that have received IPT (%)
	2012	2019	2019	2019	2019	2019	2019	2019	2019
South Africa	1160 (809-1580)	615 (427-835)	24 (14-34)	357 (248-486)	38 (36-40)	62 (25-115)	358 (286-429)	71 (65-75)	69 (55-82)
Kenya	471 (288-698)	267 (163-396)	4.2 (1.8 - 7.4)	70 (43-104)	37 (21-58)	24 (15-36)	160 (128-192)	75 (66-87)	82 (66-87)
India	234 (121-384)	193 (132-266)	9 (5.3-14)	5.2 (3.6-7.2)	32 (30-34)	0.69 (0.4-1)	158 (126-190)	60 (48-72)	45 (36-54)
Brazil	44 (38-51)	46 (39-53)	1.2 (0.9-1.5)	5.1 (4.3-6)	2.3 (2.2-2.4)	0.87 (0.65-1.1)	40 (32-48)	69 (48-90)	10 (8-12)

Table S4 ***Calibration targets for four selected countries***

++ For calibration purposes we use uncertainty intervals as estimated by WHO.
** Source: UNAIDS AIDSinfo Online Database [http://aidsinfoonline.org/devinfo/libraries/aspx/Home.aspx] All other data retrieved from WHO TB country profiles [https://worldhealthorg.shinyapps.io/tb_profiles/]*

Calibration of input parameters

We denote by θ the vector of input parameters, for all model inputs subject to uncertainty. For a given country, and a given parameter set θ, we followed the process described under 'Model implementation' for each country setting, to determine model projections for calibration targets described in **table S4.**

To compare these model projections with data *D*, we defined the *posterior density* $\pi(\theta)$ as:

$$\pi(\theta) \propto L(D|\theta).P(\theta), \qquad (28)$$

Where L is the likelihood of the data *D* given θ and *P* is the joint prior distribution for θ. For *P*, we took independent uniform distributions over the ranges shown in **Additional file 2 table of parameters S2.** The likelihood L was constructed as follows. For a given country, we fitted a beta distribution for proportions, and a log-normal distribution to all other calibration parameters in **table S4**. In particular, we determined the mean and variance of these distributions in order for the 2.5th, 50th and 97.5th percentiles to match respectively the lower, mid and upper ranges of estimates. For a given parameter set θ, we then constructed the overall likelihood $\pi(\theta)$ as a product of these distributions over all calibration targets listed in

table S4. In practice we computed the logarithm of $\pi(\theta)$, thus taking the sum of the logarithms of each of the probability densities involved.

With $\pi(\theta)$ thus defined, we sampled the posterior density using a Markov Chain Monte Carlo approach. In brief, this approach implements a random walk through the space of parameter values θ to obtain an unbiased sample of the posterior density. We implemented the 'adaptive' MCMC algorithm first introduced by *Haario et a*l[32], which incorporates a dynamic covariance matrix to adjust endogenously the scale of 'jumps' in proposals for each of the parameter values. For the set of parameter values thus obtained, we took every tenth element to reduce autocorrelation, thus yielding an 'ensemble' of parameters $\theta_1, \theta_2, \ldots$; This ensemble captures simultaneously the uncertainty in the parameter inputs, as well as in the calibration data. Then, to estimate uncertainty in a given simulated output Γ (e.g. in the reduction of incidence with a given coverage of intervention), we simulated this output Γ_i for every θ_i. We finally estimated uncertainty in Γ_i by determining its 2.5th, 50th and 97.5th percentiles.

<u>*Calibration results*</u>

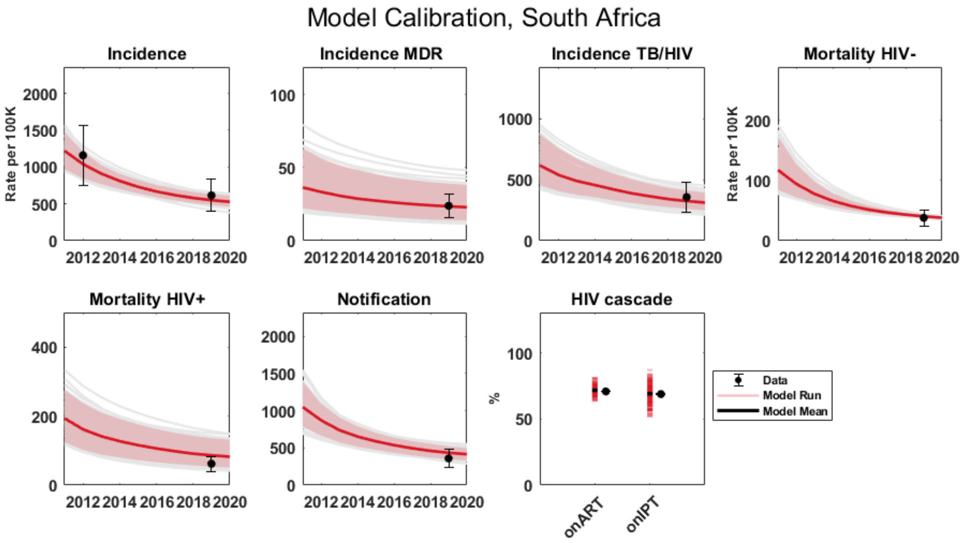

Figure S3 ***Model calibration results to epidemiological targets in South Africa***

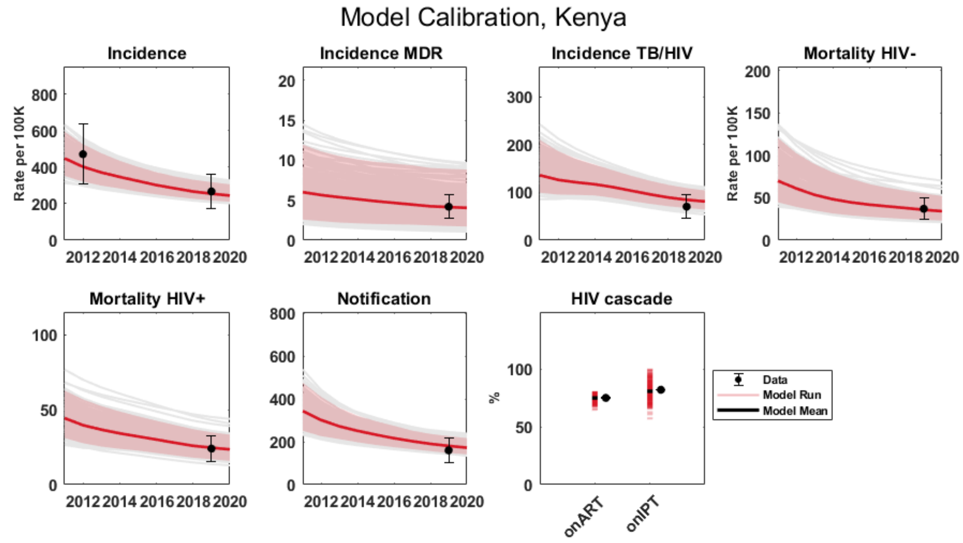

Figure S4 ***Model calibration results to epidemiological targets in Kenya***

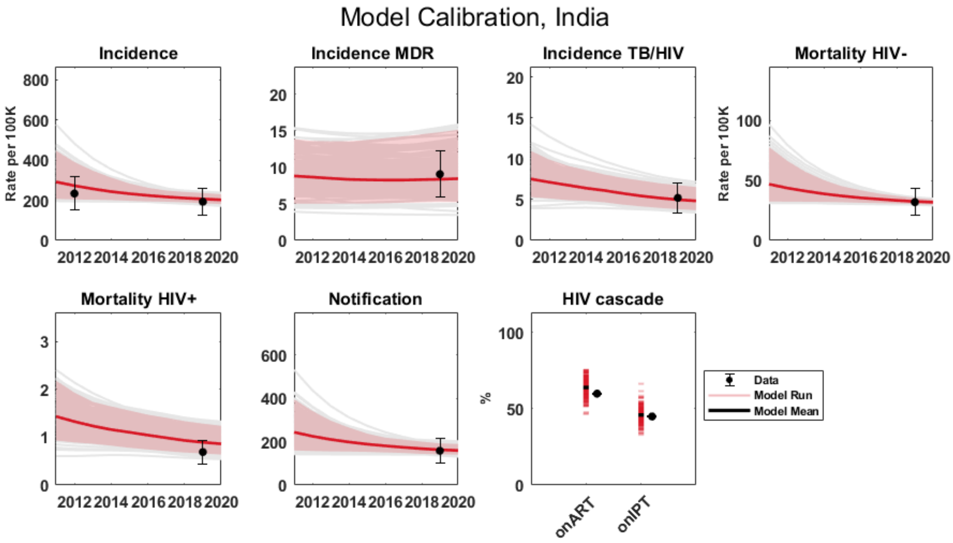

Figure S5 ***Model calibration results to epidemiological targets in India***

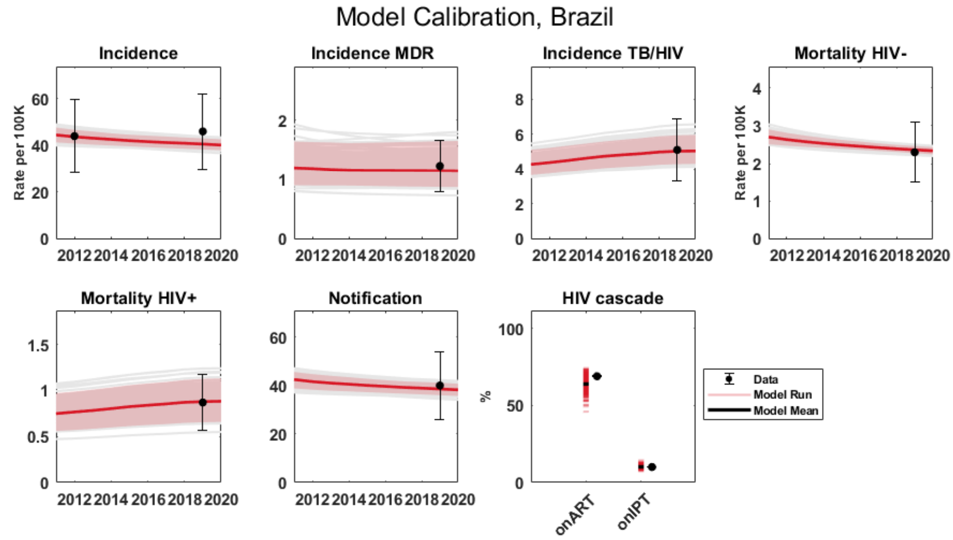

Figure S6 ***Model calibration results to epidemiological targets in Brazil***

MCMC diagnostics

Convergence was assessed visually by inspecting the trace plots of the calibrated parameters and also through estimation of the Gelman –Rubin [33] convergence diagnostic , computed as follows:

$$\hat{R} = \frac{\hat{V}}{W}, \qquad (29)$$

Where $\hat{V}$ is the posterior variance estimate of the combined chains and W is the within-chain variance. If the chains have converged to the target posterior distribution, then $\hat{R}$ (also known as the scale reduction factor) should be close to 1. As a rule of thumb, values below 1.1 are

typically considered to indicate convergence. The full trace of this diagnostic also allows us to establish what should be the size of the burn-in phase.

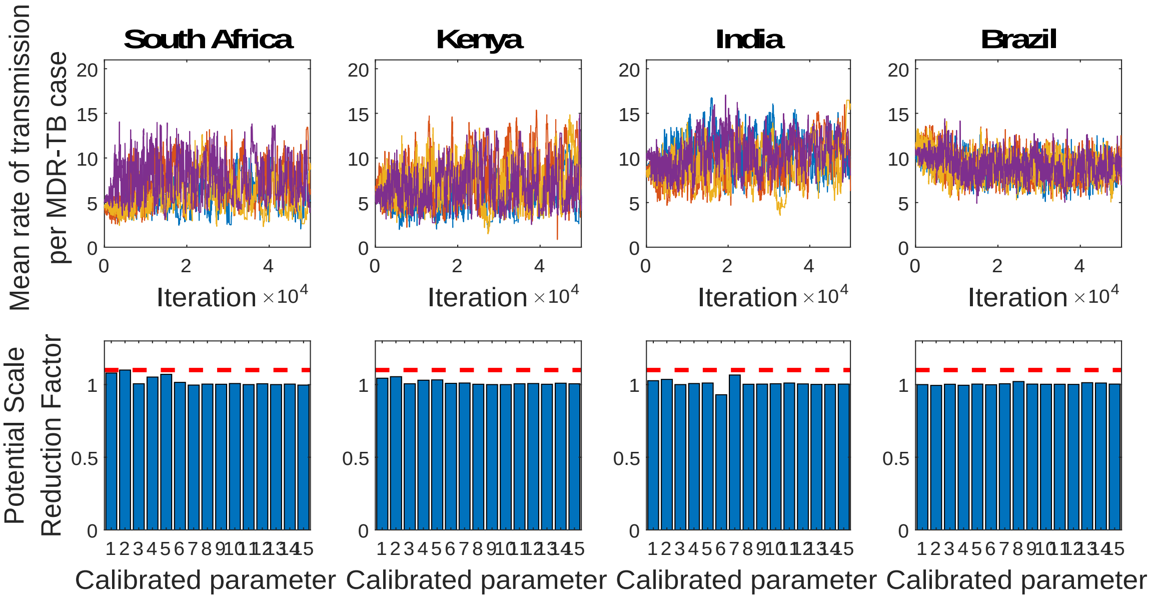

Figure S7 ***MCMC diagnostics*** *In upper row, trace plots for the posterior values of a selected calibrated parameter (Mean rate of transmission of MDR-TB). In lower row, Potential Scale Reduction Factor as a measure of autocorrelation (blue bars) and a threshold (red dashed line) of 1, indicating that burning and thinning of chains removed autocorrelation.*
