# Supplementary material for: Prioritising attributes for tuberculosis preventive treatment regimens: a modelling analysis
Source: BMC Med. 2022 May 18;20:182. doi: 10.1186/s12916-022-02378-1 (PMC9115962; doi:10.1186/s12916-022-02378-1)
Supplement: Supplementary file 5 — Additional file 5. Translating mechanisms of protection to efficacy. Table S5. Mechanistic parameters of efficacy. Fig. S8. Simulation of cohort efficacy. Fig. S9. Scatter plots of regimen properties. Fig. S10. Correlations between mechanistic parameters. [file 12916_2022_2378_MOESM5_ESM.pdf]

Additional file No. 5: Translating mechanisms of protection to efficacy

Translating mechanisms of protection to efficacy

As discussed in the main text, we developed the model of preventive treatment in such a way as to capture a range of possible mechanisms of future regimens, ranging from bacteriological cure (100%, permanent reduction in incidence in the absence of reinfection) to non-curative protection (partial reduction in incidence, for a given duration post regimen completion). As these mechanisms are unobservable with current assays, we developed the following approach to quantify their implications for regimen efficacy, as would be measured in trial conditions.

When performing Latin hypercube sampling (LHS) amongst all regimen properties as described in the main text, we also drew from the ranges for mechanistic parameters listed in **Table S5.**

**Property**	**Lower bound**	**Upper bound**
**Proportion cure**: amongst patients completing regimen, proportion having 100%, permanent reduction in incidence in the absence of reinfection	0	100%
**Strength of non-curative protection**: amongst patients completing regimen and not being cured, reduction in incidence compared to untreated, TB-infected individuals	0	100%
**Durability of non-curative protection**: following regimen completion, the duration for which non-curative protection persists (months)	0	120

Table S1 ***Assumed ranges for mechanistic parameters governing the pharmacokinetic action of a preventive regimen****. All parameters relate to outcomes for those successfully completing the regimen; further regimen properties described in Table 2 in the main text (e.g. ease-of-adherence) capture the implications of regimen implementation in programmatic conditions.*

For a given set of mechanistic parameters, we then defined a reduced form of the governing equations listed in section 1, in the following way:

- Extract equations 1 – 16 listed in Additional file1.
- Set both the force-of-infection and birth rate to zero, in order to simulate a cohort undergoing preventive treatment
- As initial conditions, take the model solution as of 2018 for the number of TB-infected individuals who are household contacts of notified cases, and who are PLHIV. Set all other state variables to zero.

By simulating this system forward in time, we therefore simulated the incidence that would be expected in these cohorts, when followed up over a period of two years. Assuming 100% adherence to represent trial conditions, we compared simulated cumulative cohort incidence with and without preventive therapy (Figure S8). By calculating the risk ratio between the two arms, we finally estimated regimen efficacy, resampling as necessary from Table S5 to yield efficacy estimates lying in the range between ‘minimal’ and ‘optimal’ scenarios listed in Table 2 in the main text.

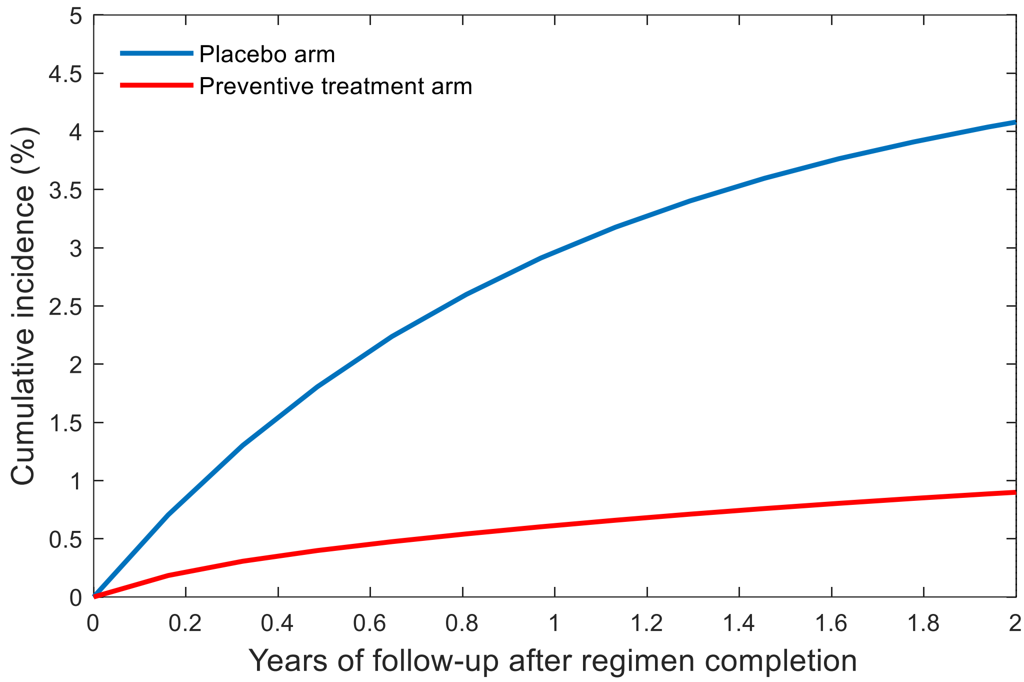

Figure S8 ***Example of regimen efficacy by comparing simulated cohorts****. In this example, the regimen is assumed to effect bacteriological cure in 50% of household contacts; and amongst the remainder, to reduce the incidence hazard by 70% for a duration of 52 months. Solid lines show cumulative incidence over a two-year followup, that would be expected from a cohort receiving this preventive treatment (red) vs one receiving placebo (blue). Overall, this regimen is estimated as having 79% efficacy, noting that efficacy is estimated as 1- relative risk at 2 years.*

Bivariate associations between regimen properties and impact

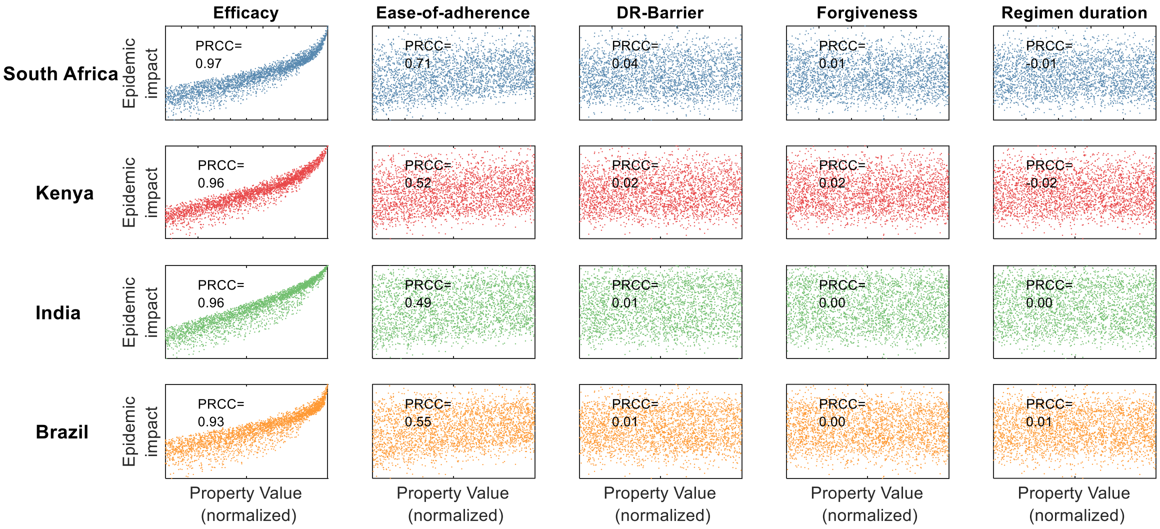

Figure S9 ***Scatter plots of individual regimen properties against impact (percent reduction in cumulative incidence between 2020 and 2035)****. The text annotation inside each panel shows the corresponding value of the partial rank correlation coefficient, as plotted in figure 3 in the main text.*

Correlation of mechanistic parameters of Efficacy

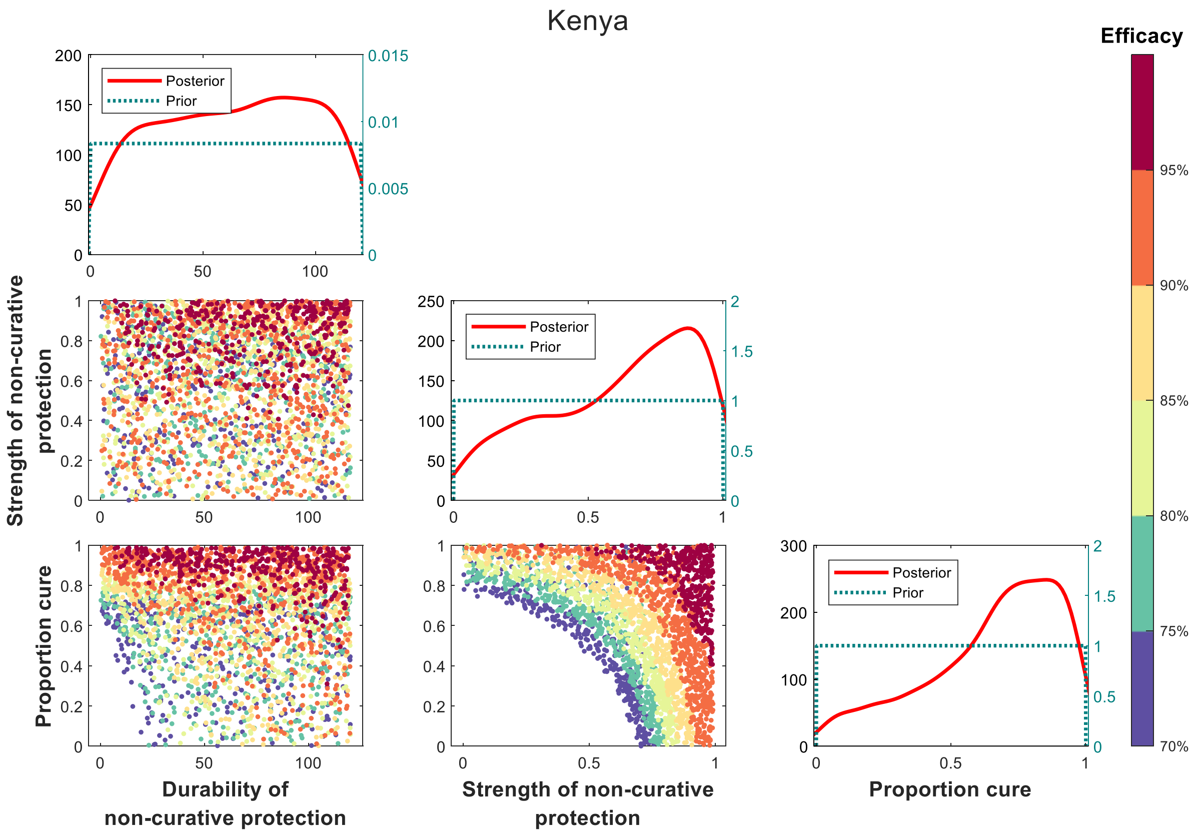

Figure S10 ***Matrix of mechanistic parameters for efficacy,*** *taking Kenya as an illustrative example (other countries show qualitatively similar results). In this matrix, diagonal plots show the marginal distributions for each mechanistic parameter. 'Prior' distributions (in blue, right-hand y-axes) show the uniform ranges that parameters were sampled from, while 'posterior' distributions (in red, left-hand y-axes) show the frequency density of points that provided efficacy values within the ranges shown in Table 2. Although all posterior densities show a right skew, this is most pronounced for the strength of non-curative protection, and for the proportion cured. Off-diagonal plots show pairwise relationships between sampled mechanistic parameters, with the colour bar indicating the corresponding level of estimated regimen Efficacy. Results suggest that the two-way parameter combination that is most influential for regimen efficacy is the combination of proportion cure, and the strength of non-curative protection (bottom left plot).*
