# Supplementary material for: Prioritising attributes for tuberculosis preventive treatment regimens: a modelling analysis
Source: BMC Med. 2022 May 18;20:182. doi: 10.1186/s12916-022-02378-1 (PMC9115962; doi:10.1186/s12916-022-02378-1)
Supplement: Supplementary file 6 — Additional file 6. Epidemiological impact of TPT by country. Fig. S11. Incidence curves under different TPT scenarios. Table S6. Summary of epidemiological impact by country. [file 12916_2022_2378_MOESM6_ESM.pdf]

Additional file No. 6: Epidemiological impact of TPT by country

Country-specific impact of preventive regimens

Here we present the country specific simulated trajectories of incidence in the four countries of study.

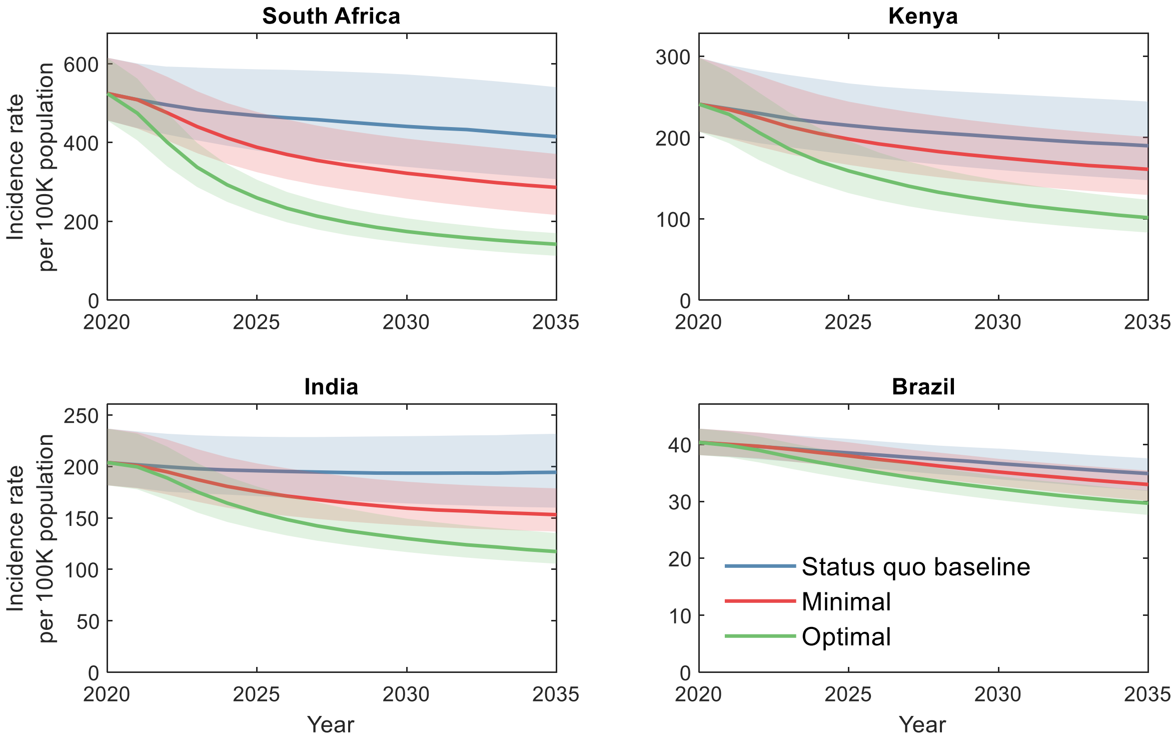

*Figure S11. **Projected epidemiological impact of future TB preventive regimens** when scaled up to cover all PLHIV undergoing HIV treatment and all household contacts of notified TB patients (see Table 2 in main text for the characteristics of minimal and optimal regimens). Blue curves show a 'status quo' comparator, where current coverage of 6H and current TB services are assumed to continue indefinitely. Red and green curves respectively show the impact of minimal and optimal regimens. Coloured, shaded regions show 95% credible intervals by Bayesian simulation. Overall impact estimates (percent reductions in cumulative cases) are shown in Table S6. Additional file 7 contains results with respect to the alternative comparator of 6H scale-up.*

Country	Minimal regimen		Optimal regimen	
	PLHIV only (%)	PLHIV + household contacts (%)	PLHIV only (%)	PLHIV + household contacts (%)
**Brazil**	1.2 (0.6-1.7)	4.2 (2.5-5)	5.4 (4.2-6.7)	10.3 (7-12)
**India**	0.3 (0.2-0.4)	13 (10-15.5)	1.1 (0.8-1.4)	21 (17-25.6)
**Kenya**	4 (3.4-5.4)	13.7 (10.6-16)	16 (13-20)	33 (30-36.3)
**South Africa**	6.7 (5-8.5)	18(14-21.2)	32 (27.3-36.5)	45 (40.4-50)

*Table S6. **Modelled epidemiological impact of future preventive treatment regimens in the four focal countries in this analysis**. Shown are model estimates for the percent reduction in cumulative incidence between 2020 and 2035, when assuming that the regimen is rolled out in each country to cover all PLHIV and all-age household contacts of notified TB cases. Numbers show median estimates, with 95% Bayesian credible intervals shown in brackets. As a comparator, we assume continued use of 6H, at current levels of coverage. 'Minimal' and 'optimal' regimens are as defined in Table 2 in the main text. Additional file 7 for corresponding results, under an alternative comparator where 6H is be scaled up to the same levels of coverage as assumed for a future preventive regimen.*
