# Supplementary material for: Prioritising attributes for tuberculosis preventive treatment regimens: a modelling analysis
Source: BMC Med. 2022 May 18;20:182. doi: 10.1186/s12916-022-02378-1 (PMC9115962; doi:10.1186/s12916-022-02378-1)
Supplement: Supplementary file 7 — Additional file 7. Further sensitivity analysis: Tables S7-S8 and Fig. S12. Results with 6H scale-up as alternative comparator. Fig. S13. Alternative optimisation order of regimen properties. Table S9 and Fig. S14. Sensitivity analysis on forgiveness thresholds. Table S10. Sensitivity analysis on efficacy of regimen on Rif resistant TB. Fig. S15 and Table S11. Widened ranges for regimen properties. Fig. S16. PRCCs assuming lower ease of adherence. Fig. S17. Effect of regimen duration on epidemiological impact. Fig. S18. PRCCs for a Rifampicin-sparing regimen. [file 12916_2022_2378_MOESM7_ESM.pdf]

Additional file No. 7: Further sensitivity analysis

Alternative comparator scenario: 6H scale-up

In the main text we assessed regimen impact against a 'status quo' comparator, assuming current levels of 6H coverage to continue indefinitely in future. Such a scenario reflects he pragmatic realities of current uptake of WHO guidelines for preventive therapy, which has so far seen only limited rollout in household contacts. To the extent that future regimens are shorter, safer and simpler than 6H, we would expect these factors to facilitate the increased uptake of preventive therapy. However, to control for coverage levels in our analysis, it is also helpful to evaluate impact under an alternative comparator, where 6H coverage is scaled up to the same extent as future preventive treatment. Properties of 6H can be found in table S7.

Regimen property	How it is quantified	Regimen	Source
		6H	
**Regimen duration**	Duration of administration of the regimen (months)	6 months	(12)
**Efficacy against drug-sensitive TB (modelled as emergent property of 'hidden' mechanistic properties including proportion bacteriologically cured, and strength and durability of non-curative protection)**	Efficacy measured as reduction in incidence among those with TB infection that would be observed under trial conditions at two-year post-regimen followup, in a cohort receiving the regimen vs a hypothetical cohort receiving placebo (see Methods for further technical details of cohort modelling) (i)	60%	(22)
**Ease-of-adherence**	Proportion successfully completing the regimen under programmatic conditions (iii)	70%	Assumption
**Forgiveness**	Amongst those completing at least half of the regimen before interrupting, the proportion that nonetheless have the same outcomes as those completing the full regimen (iv)	25%	
**Barrier to resistance**	Amongst those having rifampicin-sensitive infection, the proportion that do not develop resistant infection as a result of preventive treatment	100%	Assumption: INH only regimens do not develop Rif resistance

Table S1 ***Modelled regimen properties for 6H***

Table S8 below shows impact estimates relative to this comparator scenario, while Figure S10 shows estimates for PRCCs of future regimen properties, illustrating findings that remain qualitatively consistent with those presented in Figure 3 in the main text.

Country	Minimal regimen		Optimal regimen	
	PLHIV only (%)	PLHIV + household contacts (%)	PLHIV only (%)	PLHIV + household contacts (%)
**Brazil**	2.2 (1.8 - 2.7)	4.3 (3.5-5)	6.3 (4.9 - 7.5)	10.2 (7.7 - 11.5)
**India**	0.3 (0.2 – 0.4)	8.0 (5.8 - 10)	1.1 (0.9 – 1.5)	20.2 (17.5 - 22.7)
**Kenya**	4.2 (-3.2 - 5)	8.6 (6.8 – 10.3)	16.4 (13.2 - 20.1)	28.8 (24.8 - 34.4)
**South Africa**	8.8 (6.4 – 11.2)	12 (9.4 – 14.6)	33.4 (28.7 - 38)	41 (37 – 44.6)

Table S2 ***Epidemiological impact relative to a '6H scale-up' comparator scenario****, in which coverage of 6H is scaled up to the same levels as those assumed for the implementation of future regimens.*

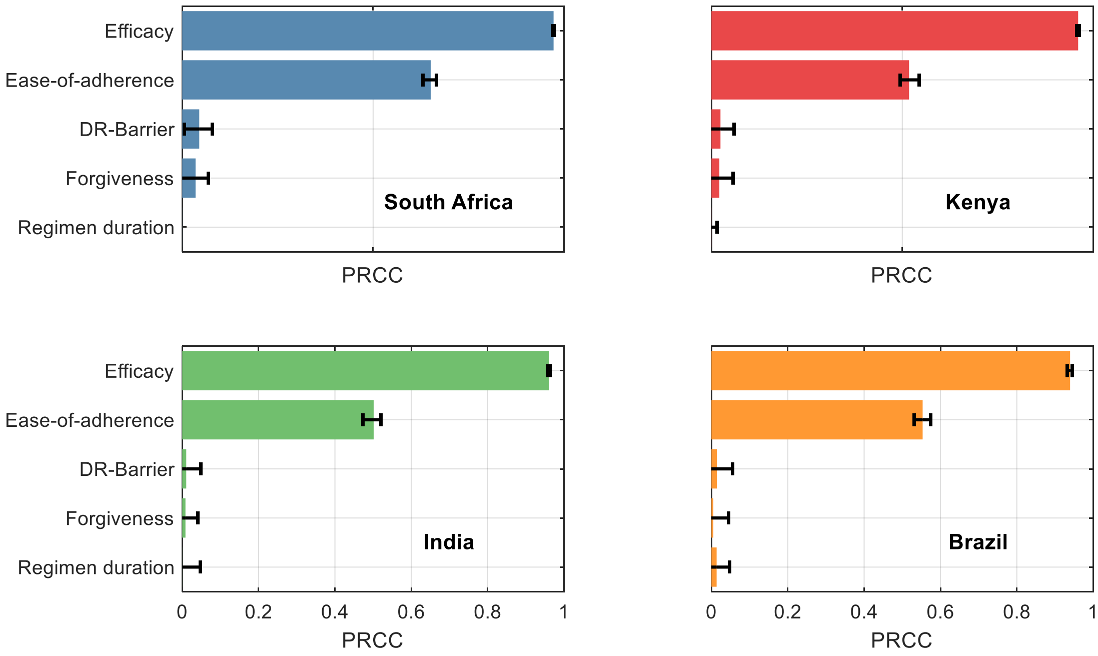

Figure S12 ***PRCCs for epidemiological impact of future regimens****, when evaluated against a '6H scale-up' comparator scenario.*

Examining the role of the order of optimisation in epidemiological impact

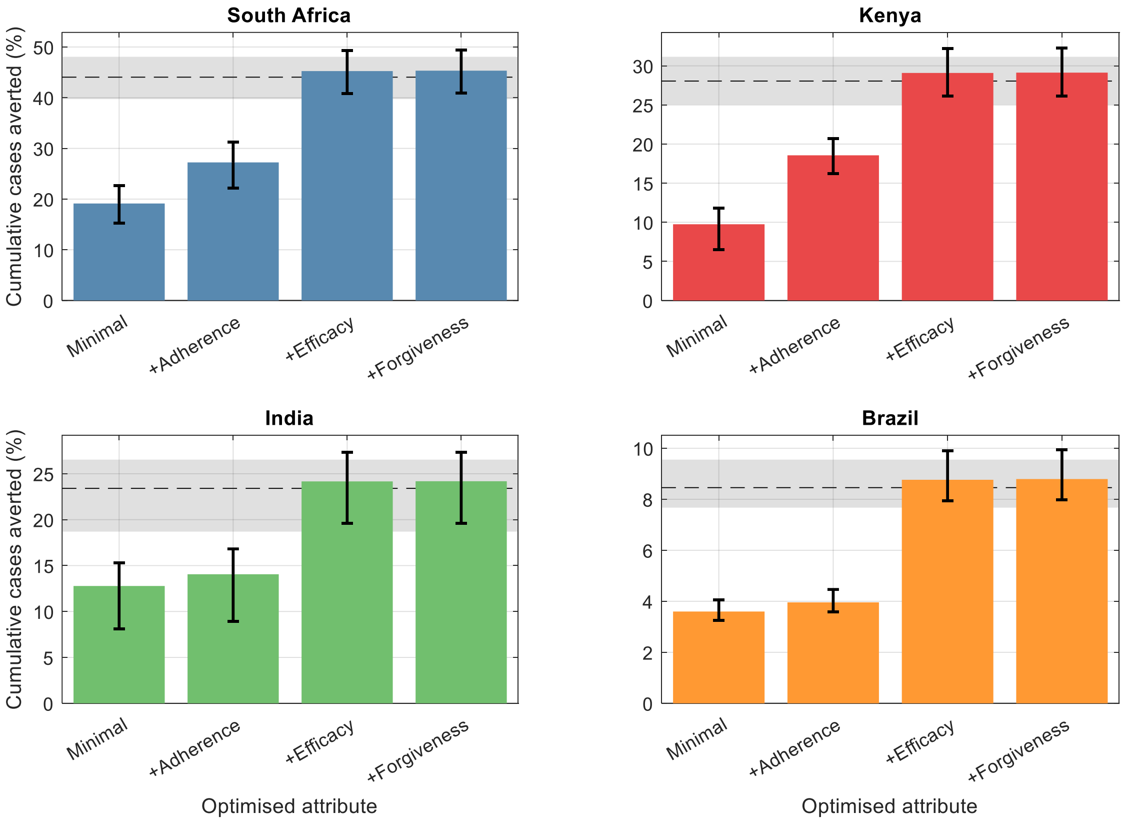

Figure S13 ***Incremental contribution of regimen properties to epidemiological impact when optimising adherence first.*** *Similar to Figure 3 in the main text, this figure shows the epidemiological impact of sequential "optimisation" of regimen properties. In this alternative analysis, ease-of-adherence is optimised first, followed by Efficacy and Forgiveness (consistent with ongoing regimen developments, yielding shorter, simpler regimens that are non-inferior to 6H). Results show the importance of adherence when improved independently, with gains in impact of ~26% from baseline for South Africa and ~50% in Kenya, and more moderate gains (~10%) in India and Brazil. This difference across settings reflects a high background transmission risk in Kenya and South Africa.*

Assumed threshold for forgiveness

In the main text, we assumed that those completing less than half the regimen would have no protective benefit and that, amongst those completing at least half the regimen before interrupting, a proportion f (equated with 'forgiveness') would have similar outcomes to those successfully completing the regimen. Thus, we assumed a threshold of 50% completion that must be satisfied, in order for forgiveness to apply. The following results show sensitivity analyses under alternative assumptions for this threshold. **Table S10** shows model findings for epidemiological impact in each country, while **Figure S14** shows the influence of each regimen property on incidence reductions, both illustrating results consistent with those presented in the main text.

	**25% Forgiveness threshold**		**75% Forgiveness threshold**	
**Regimen**	Minimal regimen	Optimal regimen	Minimal regimen	Optimal regimen
**Brazil**	3 (2.2-3.5)	8 (6.5-10)	2.3(1.4-3)	8.6(6.8-10)
**India**	13 (9-16)	24(19-27)	12 (6-14)	24(19-27)
**Kenya**	10 (8-12)	27 (24-30)	9 (5-11)	31(28-34)
**South Africa**	16 (12.5-19.7)	42 (37.3-47)	21 (16-24)	48 (43-52)

Table S9 ***Epidemiological impact (percent reductions in cumulative incidence between 2020 – 2035)*** *under different minimum thresholds for the amount of a regiment that needs to be administered, for forgiveness to apply. In the main text we assume a threshold of 50% regimen completion.*

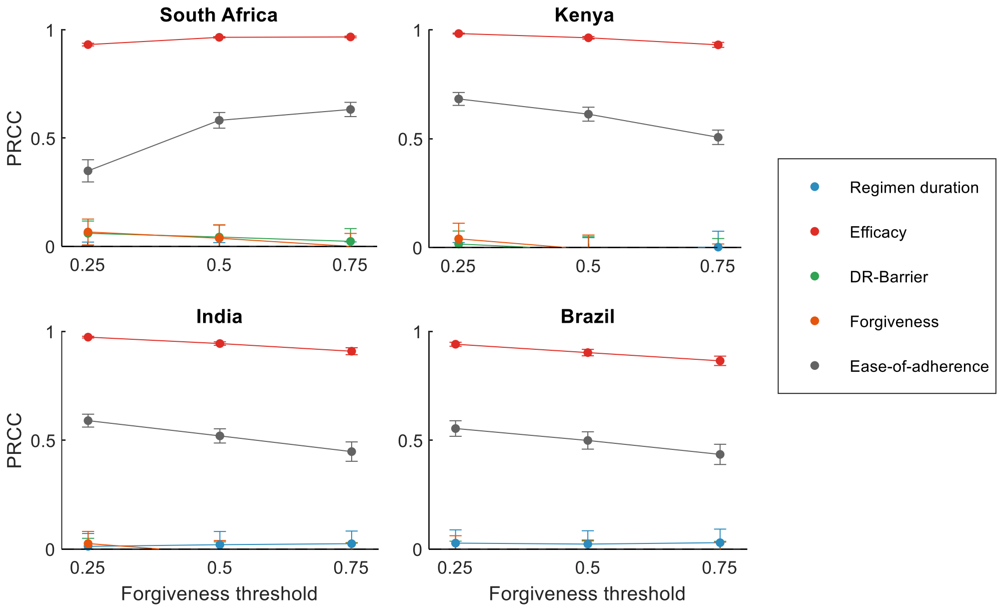

Figure S14 ***Sensitivity analysis for the effect of forgiveness threshold, on results for incidence-reducing influence of different regimen properties****. Shown are PRCCs for individual regimen properties, vs incidence impact. These figures are an alternative representation of PRCCs to the bar charts shown in Figure 3 in the main text; they allow comparison across different assumptions for the forgiveness threshold (shown on the x-axis), with each colour representing a different regimen property as listed in the legend.*

Regimen efficacy against infection with rifampicin resistant TB

For results presented in the main text, we assumed that a future preventive regimen would be implemented amongst all contacts, regardless of the drug sensitivity status of the index case; we further assumed that such a regimen would have half the efficacy against RR-TB, as against drug-sensitive TB. Table S10 shows impact estimates under alternative assumptions for relative efficacy, showing similar findings to those reported in Table 3 in the main text.

	**25% Effect on DR strains**		**75% Effect on DR strains**	
**Regimen**	Minimal regimen	Optimal regimen	Minimal regimen	Optimal regimen
**Brazil**	2.6 (1.8-3.3)	8.6 (6.7-10)	2.7 (1.8-3.3)	8.6 (6.6-10)
**India**	12.7 (8-15)	24 (19-28)	12.5 (7.7-15)	24 (19-29)
**Kenya**	9.8 (6.7-11.7)	29 (26-32)	9.6(6.6-11.6)	29.4(26-33)
**South Africa**	19(15-22)	44 (40-49)	19 (14-22)	45 (40-49)

Table S10 ***Epidemiological impact for varying effect of PT regimens on rifampicin-resistant strains***

Widened ranges for regimen properties

Parameter ranges presented in Table 2 in the main text were elicited from expert opinion; model results may well depend on this choice of ranges. To examine sensitivity to these assumptions, Figure S15 shows model results with respect to widened ranges for each of the regimen properties. In order to quantify the gradient of the black lines shown in this Figure, Table S11 shows coefficients estimated from a univariate regression, and from a simple multivariate regression (using linear models in both cases). Coefficients show results consistent with those illustrated in Figure 2 in the main text: that is, a dominant role of efficacy, and an important secondary role of ease-of-adherence.

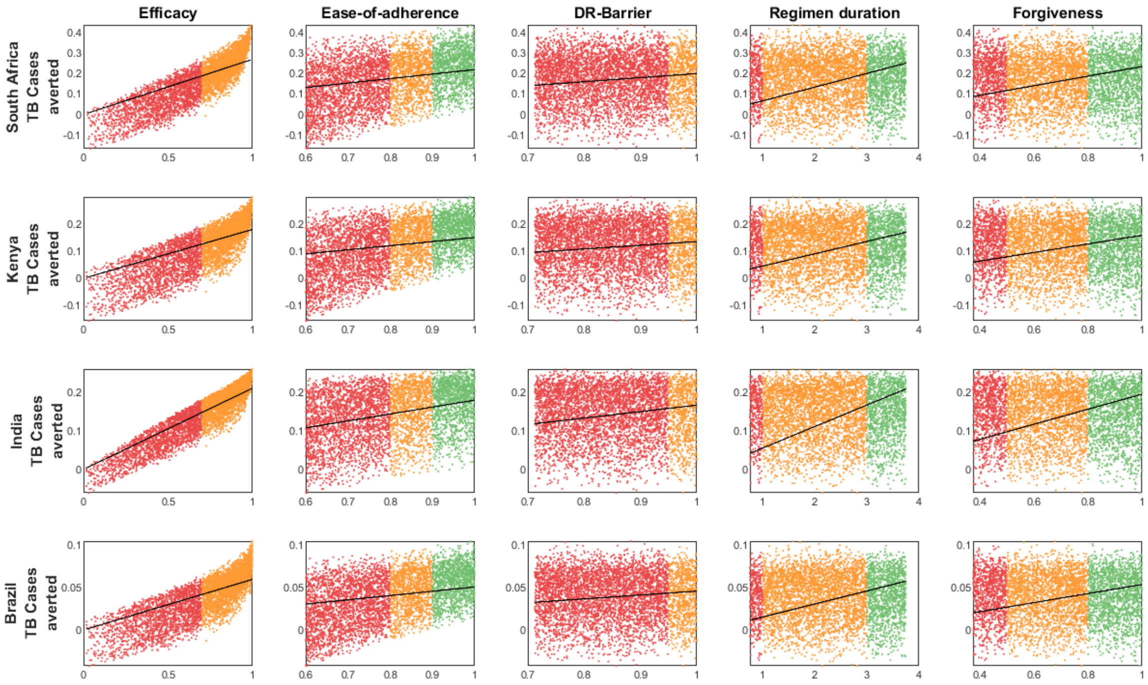

Figure S15 ***Results of widened parameter ranges for the regimen properties****. Figures show scatter plots of property value vs. epidemiological impact (TB cases averted up to 2035). Each column reflects one regimen property, while rows display countries. Dots in orange represent model simulations within the ranges specified in Table 2 in the main text. Dots in red and green show, respectively, model simulations from parameters below and above this range. Solid black lines show best fits from univariate linear regressions (see Table S11 for coefficients with uncertainty intervals, along with estimates from a simple multivariate regression).*

**Regimen Properties**	**Univariate Regression coefficient (95% CI)**	**Multivariable Regression coefficient (95% CI)**
Efficacy	0.437 (0.43-0.44)	0.435 (0.43-0.438)
Ease-of-adherence	0.407 (0.39-0.43)	0.399 (0.392-0.405)
DR Barrier	0.031 (0.01-0.06)	0.038 (0.028-0.047)
Regimen Duration	0.003 (-0.02-0.03)	0.0008 (-0.0005-0.0017)
Forgiveness	0.001 (-0.02-0.03)	0.006 (0.0019-0.010)

Table S11 ***Linear regression analysis of simulated regimen properties in Kenya.*** *Shown are coefficients arising from regressing TB cases averted against each of the regimen properties listed in the left-hand column. Second and third columns show results, respectively, of univariate and multivariate regressions.*

PRCCs under alternative Ease-of-adherence assumptions

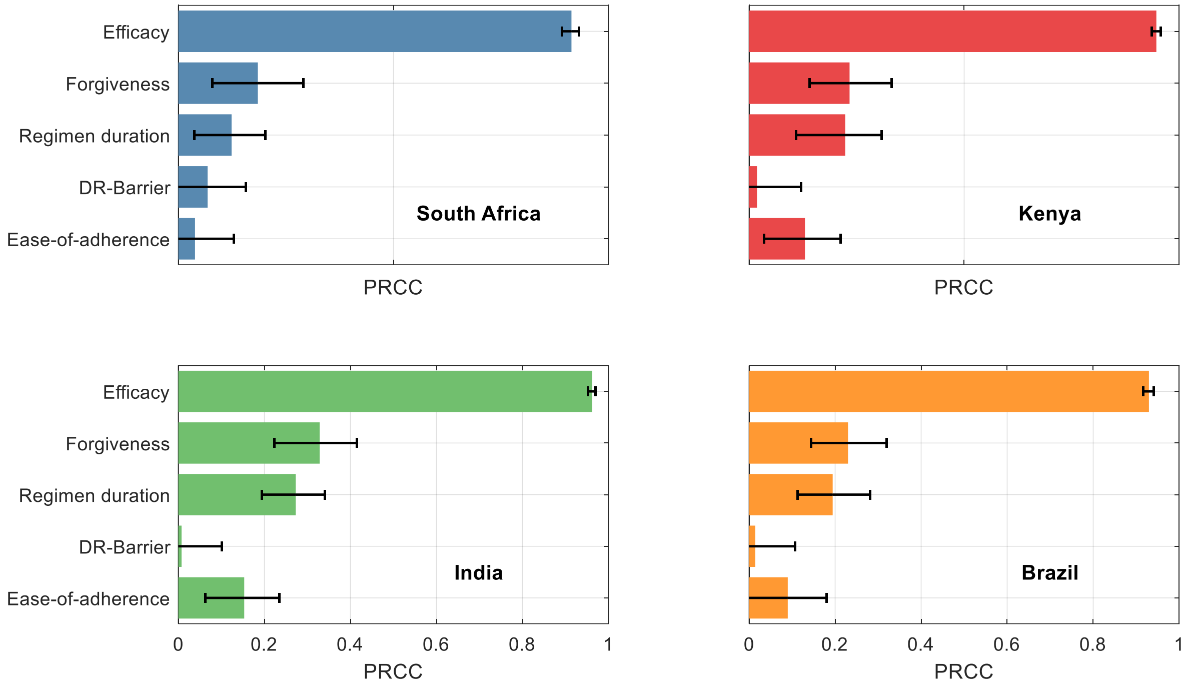

Figure S16 ***Results of PRCC with alternative ranges of 40 – 42% for Ease-of-adherence****. As in Figure 2 in the main text, shown are partial rank correlation coefficients (PRCCs) between each regimen property listed in Table 2, and the epidemiological impact (cumulative incidence reduction between 2020 and 2035) of a regimen covering all PLHIV on ART, as well as all household contacts of notified cases. In this alternative analysis, we aim to construct conditions favouring the importance of forgiveness, as follows: (i) the range for ease-of-adherence, or equivalently regimen completion, is restricted to 40% - 42%,and (ii) the proportion of the regimen needing to be completed before forgiveness can apply is lowered to 5% (compared to 50% in the main text). The Figure shows that under these alternative conditions, forgiveness replaces ease-of adherence as the second more important property, although efficacy remains the most important. The apparent reduction in the importance of ease-of-adherence, relative to the results shown in Figure 2, is likely an artefact of the reduced range of values [40 – 42%] assumed here.*

Effect of regimen duration on overall effectiveness

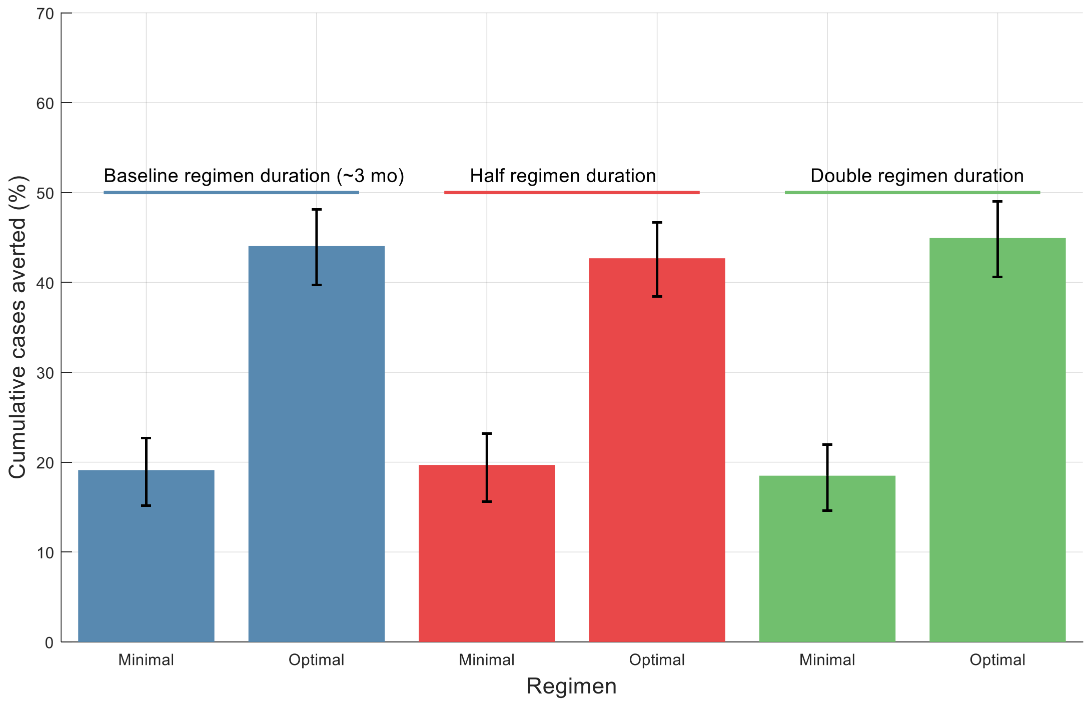

Figure S17 ***Influence of regimen duration on epidemiological impact****. Here, we show incidence reductions in South Africa as an example. Bars show cumulative cases averted over the simulation period for minimal and optimal regimens, for regimens with baseline duration of 3 months (Blue), half this duration (red) and double this duration (green). Error bars show 95% Credible intervals around the posterior mean. Results illustrate that regimen duration has little substantial effect on the overall impact of preventive therapy even in the context of South Africa, the setting with a highest background TB transmission rate (i.e., force of infection). As noted in the main text, here we are* not *addressing the adherence benefits of shorter, simpler regimens, that are captured separately under the 'ease-of-adherence' attribute.*

Rifampicin-sparing regimens

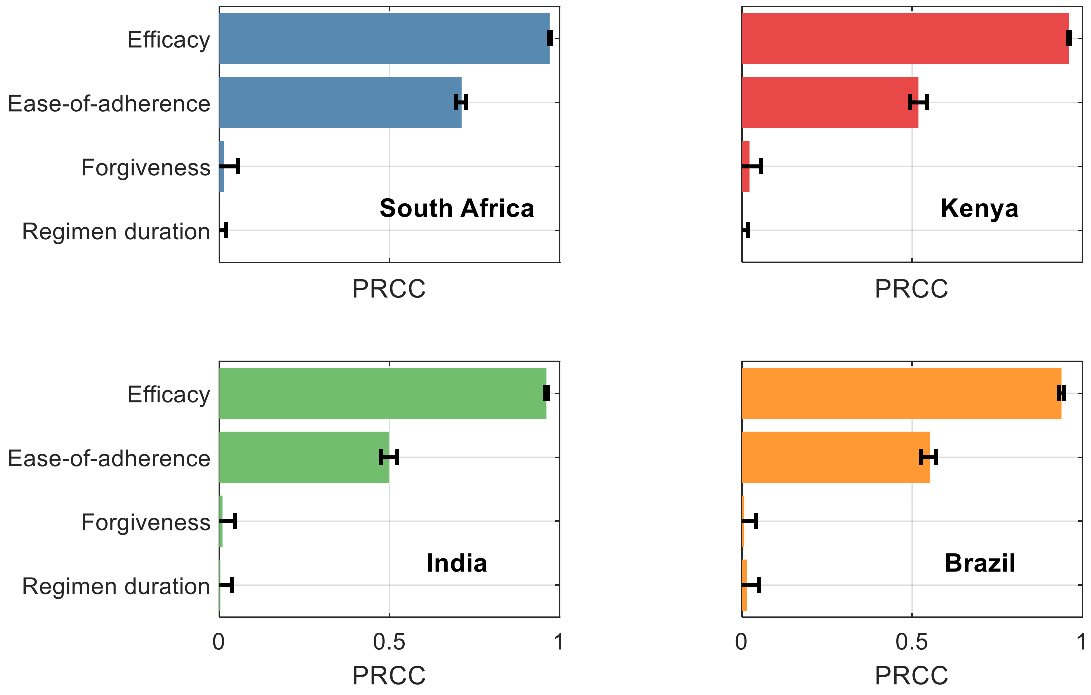

Figure S18 ***Influence of regimen properties on incidence reductions, for Rifampicin-sparing regimens.*** *Such regimens would have essentially zero risk of inducing rifampicin resistance: results in the figure thus show partial rank correlation coefficients where (amongst the regimen properties listed in Table 2) the barrier to developing RR-TB is held at 100%.*
